# Breast cancer anti-estrogen resistance 3 inhibits transforming growth factor β/Smad signaling and associates with favorable breast cancer disease outcomes

**DOI:** 10.1186/s13058-014-0476-9

**Published:** 2014-12-13

**Authors:** Jimin Guo, Lucie Canaff, Charles Vincent Rajadurai, Nadège Fils-Aimé, Jun Tian, Meiou Dai, Juliana Korah, Manuel Villatoro, Morag Park, Suhad Ali, Jean-Jacques Lebrun

**Affiliations:** 1 Division of Medical Oncology, Department of Medicine, McGill University Health Center, H7 Royal Victoria Hospital, 687 Pine Avenue West, Montreal, H3A 1A1 Quebec Canada; 2 Rosalind and Morris Goodman Cancer Center, 1160 Pine Avenue West, Montreal, H3A 1A3 Quebec Canada; 3 Division of Hematology, Department of Medicine, McGill University Health Center, H7 Royal Victoria Hospital, 687 Pine Avenue West, Montreal, H3A 1A1 Quebec Canada

## Abstract

**Introduction:**

This study helps to define the implications of breast cancer anti-estrogen resistance 3 (BCAR3) in breast cancer and extends the current understanding of its molecular mechanism of action. BCAR3 has been shown to promote cell proliferation, migration and attachment to extracellular matrix components. However, in a cohort of metastatic breast cancer patients who received tamoxifen treatment, high BCAR3 mRNA levels were associated with favorable progression-free survival outcome. These results suggest that, besides its established roles, BCAR3 may have additional mechanisms of action that regulate breast cancer aggressive phenotype. In this study, we investigated whether BCAR3 is a novel antagonist of the canonical transforming growth factor β (TGFβ) pathway, which induces potent migration and invasion responses in breast cancer cells.

**Methods:**

We surveyed functional genomics databases for correlations between BCAR3 expression and disease outcomes of breast cancer patients. We also studied how BCAR3 could regulate the TGFβ/Smad signaling axis using Western blot analysis, coimmunoprecipitation and luciferase assays. In addition, we examined whether BCAR3 could modulate TGFβ-induced cell migration and invasion by using an automated imaging system and a confocal microscopy imaging–based matrix degradation assay, respectively.

**Results:**

Relatively low levels of BCAR3 expression in primary breast tumors correlate with poor distant metastasis-free survival and relapse-free survival outcomes. We also found a strong correlation between the loss of heterozygosity at *BCAR3* gene alleles and lymph node invasion in human breast cancer, further suggesting a role for BCAR3 in preventing disease progression. In addition, we found BCAR3 to inhibit Smad activation, Smad-mediated gene transcription, Smad-dependent cell migration and matrix digestion in breast cancer cells. Furthermore, we found BCAR3 to be downregulated by TGFβ through proteasome degradation, thus defining a novel positive feedback loop mechanism downstream of the TGFβ/Smad signaling pathway.

**Conclusion:**

BCAR3 is considered to be associated with aggressive breast cancer phenotypes. However, our results indicate that BCAR3 acts as a putative suppressor of breast cancer progression by inhibiting the prometastatic TGFβ/Smad signaling pathway in invasive breast tumors. These data provide new insights into BCAR3’s molecular mechanism of action and highlight BCAR3 as a novel TGFβ/Smad antagonist in breast cancer.

**Electronic supplementary material:**

The online version of this article (doi:10.1186/s13058-014-0476-9) contains supplementary material, which is available to authorized users.

## Introduction

Breast tumorigenesis and progression are controlled by multiple hormone/growth factor/cytokine signaling pathways, which are ideal therapeutic targets. Targeted therapies against breast cancer, such as those aimed at estrogen receptor α (ERα) or the Her2 receptor tyrosine kinase, have shown some levels of success [1],[2]. However, clinical observations also indicate that tumors that initially respond to targeted therapies often relapse and acquire resistance to the treatments [3],[4]. Several genes, collectively named breast cancer anti-estrogen resistance (*BCAR*) genes, have been found to induce estrogen-independent cell growth in estrogen-dependent breast cancer cells [5]. Two members, BCAR1/p130Cas and BCAR3, have been found to form a complex by directly interacting with each other [6],[7]. Individual overexpression of these genes allows estrogen-dependent breast cancer cells to proliferate under the presence of tamoxifen [5],[8]. Ectopic overexpression of BCAR3 in breast cancer cells activates Src and FAK kinases, leading to p130Cas tyrosine phosphorylation and increased cell attachment to fibronectin and cell motility [7],[9]. Therefore, BCAR3 is currently considered to play a role in mediating aggressive breast cancer phenotypes. However, the authors of a previous report suggested that BCAR3 expression is correlated with favorable outcome in progression-free survival (PFS) in a cohort of ER-positive (ER+) breast cancer patients who had received tamoxifen treatment [10]. As such, BCAR3 has controversial implications in breast cancer.

During breast cancer progression, alongside the development of hormone-independent growth mechanisms, cancer cells have been shown to alter their biological response to transforming growth factor β (TGFβ) [11]. TGFβ family growth factors, through induction of cell cycle arrest and apoptosis, inhibit cell proliferation in the mammary epithelium and in well-differentiated, early-stage breast tumors [12]-[15]. These functions are lost and replaced by tumor-promoting and prometastatic responses in poorly differentiated, advanced-stage breast tumors [16]-[19]. In cancer cells representing such tumors, TGFβ transcriptionally reprograms cells to induce epithelial-to-mesenchymal transition and cell migration and invasion [19],[20]. In addition, in the stroma, TGFβ promotes local and systematic immune suppression, thereby allowing transformed cells to escape immune surveillance, further promoting tumor metastasis [19],[21],[22]. Most of these biological functions of TGFβ are attributed to a canonical signaling pathway mediated by the Smad transcription factors [23]. The binding of TGFβ to its receptors (type I and type II serine/threonine kinases) leads to the recruitment and phosphorylation of Smad2/3 and the association of Smad2/3 with Smad4. The activated Smads then collectively translocate into the nucleus, where they bind to regulatory elements on the promoter regions of their target genes to regulate gene transcription [24]. The canonical TGFβ/Smad signaling axis is central to TGFβ-mediated breast cancer cell migration and tumor metastasis. Alteration of the function of key components of the TGFβ/Smad signaling by using RNA interference or decoy ligand traps approaches impairs the formation of breast cancer metastasis in experimental models [16],[25],[26]. As transcription factors, Smad proteins are not ideal drug targets. Therefore, understanding how intracellular mechanisms regulate Smad signaling provides insights into the biology of metastatic breast cancer and into novel means of treatment and prognosis.

In this study, we define a novel regulatory pathway directly linking the TGFβ/Smad signaling axis to BCAR3. Our data highlight BCAR3 as a potent inhibitor of the TGFβ/Smad signaling pathway. We found BCAR3 to promote an interaction between Smad2/3 and p130Cas, leading to inhibition of Smad activation, Smad-mediated gene transcription and Smad-dependent cell migration and invasion in breast cancer cells. We also found BCAR3 protein levels in breast cancer cells to be controlled by TGFβ, as TGFβ treatment decreases BCAR3 expression in a Smad-dependent and proteasome-dependent manner. Our findings define a novel positive regulatory feedback loop through which TGFβ signaling further induces its effects by blocking expression of the Smad inhibitor BCAR3. Additionally, we report a true prognostic value of BCAR3 in human breast cancer. We found that loss of BCAR3 expression in primary breast tumors correlates with poor outcomes. Taken together, our study indicates that BCAR3 is a novel antagonist of TGFβ proinvasive functions in breast cancer cells, and loss of BCAR3 function correlates with poor outcomes in breast cancer patients.

## Methods

### Gene expression analysis

Using Gene Expression-Based Outcome for Breast Cancer Online (GOBO), patient outcomes (disease-free survival (DFS), relapse-free survival (RFS) and distant metastasis-free survival (DMFS)) were quarried for 10 years of data. Compiled cohorts of patients were divided as described below, based on reading from an Affymetrix BCAR3 probe (Affymetrix, Santa Clara, CA, USA) in microarrays (204032_at). In the analysis of endocrine agent-treated patients, logrank readings from the probe targeting BCAR3 were extracted from a National Center for Biotechnology Information (NCBI) Gene Expression Omnibus (GEO) dataset GDS807 and plotted into a dot plot.

### Cell culture

MCF-7, MDA-MB-231, BT-549 and SK-BR-3 cells were obtained from the American Type Culture Collection (Manassas, VA, USA) and maintained in Dulbecco’s modified Eagle’s medium (DMEM; HyClone Laboratories, Logan, UT, USA) supplemented with 10% fetal bovine serum (FBS). Inducible BCAR3 MCF-7 cells were maintained in DMEM supplemented with 10% FBS, 100 μg/ml G418 and 1 μg/ml puromycin. SUM-149PT and SUM-159PT cells were obtained from Dr. Stephen Ethier (Department of Pathology and Laboratory Medicine, Medical University of South Carolina, Charleston, SC, USA) and were maintained in F-12 nutrient mixture (HyClone Laboratories) supplemented with 5% FBS, 5 μg/ml insulin and 1 μg/ml hydrocortisone. SCP2 cells were obtained from Dr. Joan Massagué (Memorial Sloan Kettering Cancer Center, New York, NY, USA). Table 1 summarizes some basic properties of these cells, with part of the information adopted from a previous study [27].

**Table 1:** **Properties of breast cancer cell lines used in this study** ^**a**^

Cell lines	Subtype	ER	PR	Her2	p53	TGFβ effects	Other properties
MCF-7	Luminal	+	+		+/− Wild type	Growth inhibition	
MDA-MB-231	Basal B	−	−		+/+ Mutated	Migration, invasion	
SCP2	Basal B	−	−		Not tested	Migration, invasion	Metastasize to bone
BT-549	Basal B	−	−		+/+ Mutated	Migration, invasion	
SUM-149PT	Basal B	−	−		+ As per mRNA	Growth inhibition	
SUM-159PT	Basal B	−	−		+ As per mRNA	Invasion	
SK-BR-3	Luminal	−	−	Overexpress	Mutated		
MCF-7	Luminal	+	+		+/− Wild type	Growth inhibition	
MDA-MB-231	Basal B	−	−		+/+ Mutated	Migration, invasion	
SCP2	Basal B	−	−		Not tested	Migration, invasion	Metastasize to bone
BT-549	Basal B	−	−		+/+ Mutated	Migration, invasion	
SUM-149PT	Basal B	−	−		+ As per mRNA	Growth inhibition	
SUM-159PT	Basal B	−	−		+ As per mRNA	Invasion	
SK-BR-3	Luminal	−	−	Overexpress	Mutated		

^a^ER, Estrogen receptor; Her2, Human epidermal growth factor receptor 2; PR, Progesterone receptor; TGFβ, Transforming growth factor β.

### Constructs and transfection

BCAR3 small interfering RNAs (siRNAs) (#1: SASI_Hs01_00236261; #2: SASI_Hs02_00335873), p130Cas siRNA (SASI_Hs02_00345830) and scrambled control siRNA (universal negative control #2) were manufactured by Sigma-Aldrich (St Louis, MO, USA). For mock transfection, cells were transfected in the same manner, but without siRNA. Instead, an equal volume of distilled water was added to the transfection mixture in lieu of siRNA. Dr. Laurence Quilliam (Department of Biochemistry and Molecular Biology, Indiana University School of Medicine, Indianapolis, IN, USA) kindly provided the FLAG-tagged mouse AND-34 expression vector. Transfections were carried out using Lipofectamine 2000 reagent (Life Technologies, Carlsbad, CA, USA) according to the manufacturer’s instructions.

### Coimmunoprecipitation

Cells were lysed with radioimmunoprecipitation assay (RIPA) buffer containing 1% Triton X-100, protease inhibitors and phosphatase inhibitors. Total protein lysates were quantified, and lysates containing 1 mg of total protein were subjected to coimmunoprecipitation using a rabbit polyclonal antibody raised against Smad2/3 (FL425; Santa Cruz Biotechnology, Santa Cruz, CA, USA) overnight at 4°C. Lysates were then incubated with protein A Sepharose beads for 2 hours at 4°C. Beads were then washed three times with RIPA buffer, mixed with 2× SDS loading buffer, boiled for 5 minutes and subjected to SDS-PAGE.

### SDS-PAGE and Western blot analysis

Cells were lysed with RIPA buffer containing 1% Triton X-100, protease inhibitors and phosphatase inhibitors. Total protein lysates were quantified, and lysates containing 50 μg of total protein were separated by SDS-PAGE and then transferred onto nitrocellulose membranes and subjected to Western blot analysis as previously described [28]. Densitometry of Western blots was quantified using Quantity One 1-D analysis software (Bio-Rad Laboratories, Hercules, CA, USA).

To obtain nuclear extracts, cells were lysed with phosphate-buffered saline (PBS) containing 1% Nonidet P-40. The nucleus were washed in the lysis buffer multiple times and lysed with loading dye containing SDS, as described in a protocol developed by others [29].

The primary antibodies used for Western blot analysis were rabbit polyclonal Smad2/3 antibody (sc-8332; Santa Cruz Biotechnology), rabbit phospho-Smad3 antibody (9520; Cell Signaling Technology, Danvers, MA, USA), goat polyclonal BCAR3 antibody (sc-47811; Santa Cruz Biotechnology), rabbit polyclonal p130Cas antibody (sc-860; Santa Cruz Biotechnology), rabbit polyclonal USF-2 antibody (sc-862; Santa Cruz Biotechnology) and mouse monoclonal β-tubulin antibody (sc-5274; Santa Cruz Biotechnology). All corresponding secondary antibodies were purchased from Santa Cruz Biotechnology.

### Luciferase assay

Cells seeded into six-well plates were cotransfected as described above with 1 μg of (CAGA)12-lux luciferase reporter, 1 μg of β-galactosidase reporter and either siRNA (50 pM final concentration) or cDNA construct (3 μg/well). Cells were then treated with or without 200 pM TGFβ for 24 hours and used for luciferase assays as described previously [28].

### RNA extraction, reverse transcription and real-time PCR

Total RNAs were extracted with TRIzol reagent (Life Technologies) according to the manufacturer’s instructions. RNA samples were reverse-transcribed using Moloney murine leukemia virus (Life Technologies) and subjected to real-time PCR for connective tissue growth factor (CTGF), transmembrane prostate androgen induced RNA (TMEPAI) and Smad7, with ribosomal 18S RNA used as an internal control. In experiments performed to study the regulation of BCAR3 by TGFβ, CTGF was used as a positive control and glyceraldehyde 3-phosphate dehydrogenase was used as an internal control. Table 2 shows the primer sequences used.

**Table 2:** **Primer sequences**

Primer	Sequence 5′-3′
CTGF-U	GGTTACCAATGACAACGCCT
CTGF-L	TGGAGATTTTGGGAGTACGG
TMEPAI-U	CAAGCCTCCTGGTCTTTCTG
TMEPAI-L	GACCGTGCAGACAGCTTGTA
Smad7-U	TGCTCCCATCCTGTGTGTTAAG
Smad7-L	TCAGCCTAGGATGGTACCTTGG
18S-U	ATACATGCCGACGGGCACTG
18S-L	TTCGAATGGGTCGTCGCCGC
BCAR3-U	ATCTTCCAGCCCATCAACAG
BCAR3-L	TTTCTGAGGAGGTTTCCCCT
GAPDH-U	GCCTCAAGATCATCAGCAATGCCT
GAPDH-L	TGTGGTCATGAGTCCTTCCACGAT

PCRs were carried out using SsoFast EvaGreen supermix (Bio-Rad Laboratories) according to the manufacturer’s instructions. Briefly, reactions were activated at 95°C for 30 seconds and then underwent 40 cycles of amplification. Each cycle comprised 5-second denaturation at 95°C and 30-second annealing/extension at 60°C.

### Cell viability assay

Inducible MCF-7 cells were plated into 96-well plates (5,000 cells/well) and cultured in complete DMEM with or without doxycycline for 96 hours. Cells were then serum-starved with or without doxycycline. Under each condition, paired wells of cells were treated with or without 200 pM TGFβ for 72 hours. Cells were then incubated with 3-(4,5-dimethylthiazol-2-yl)-2,5-diphenyltetrazolium bromide (MTT; thiazolyl blue tetrazolium bromide) for 4 hours and gently washed with PBS. Dimethyl sulfoxide (200 μl/well) was added to suspend converted formazan, subjected to absorption reading at 570 nm.

### Confocal microscopy

Cells transfected with FLAG-tagged AND-34 were seeded onto coverslips, treated as described in the figure legends, fixed with 3.7% paraformaldehyde in PBS for 15 minutes, and then permeabilized with 0.1% Triton X-100 in PBS for 3 minutes. After blocking for 1 hour at room temperature in 2% bovine serum albumin in PBS, cells were costained with a mouse anti-FLAG antibody (1:500 dilution, M2; Sigma-Aldrich) and a rabbit anti-phospho-Smad3 antibody (1:500 dilution, 9520; Cell Signaling Technology) or Alexa Fluor 568–labeled phalloidin (Life Technologies). Cells were then stained with Alexa Fluor 488–labeled goat-anti-mouse secondary antibody (1:500 dilution; Life Technologies) and Alexa Fluor 568–labeled goat anti-rabbit secondary antibody (1:500 dilution; Life Technologies), except when phalloidin was used. Following 4′,6-diamidino-2-phenylindole counterstaining and mounting, images were taken using a × 63 oil-immersion lens objective with an LSM780 confocal microscope (Carl Zeiss, Oberkochen, Germany). Images were taken in a multitrack scanning mode at 1024 × 1024 resolution. Excitation wavelengths were set at 490 nm (argon laser) and 570 nm (helium-neon laser) to detect emission wavelengths at around 520 nm (for Alexa Fluor 488) and about 600 nm (for Alexa Fluor 568), respectively. Images were converted to 16-bit TIFF RGB format using ImageJ software (National Institutes of Health, Bethesda, MD, USA). Quantification of phospho-Smad levels was performed using ImageJ software (five images per condition). Quantification of stress fiber length was performed using ImageJ software by measuring the distance between the visible ends of fibers (five random fibers per cell, five cells per condition).

### Migration assays

Scratch-based migration assays were carried out with an IncuCyte automated imaging system (Essen BioScience, Ann Arbor, MI, USA) according to the manufacturer’s protocol. Briefly, SCP2 cells transfected with siRNA were seeded onto ImageLock 96-well plates (Essen BioScience) 1 day after transfection at a density of 50,000 cells/well. Cells were then starved overnight. Monolayers of cells were scratched using a scratching apparatus that produced strongly identical scratches in each well. Cells were then treated with 100 pM TGFβ. The IncuCyte system was programmed to obtain real-time phase-contrast images of the wounds at 12 time points. In the BCAR3 siRNA experiments, images were taken every 4 hours for 48 hours. In the double-knockdown experiments, images were taken every 3 hours for 36 hours. Cell migration was quantified and expressed as relative wound density, which indicates the ratio of sharpness of the wounded area and of the adjacent nonwounded area. The siRNA experiments in which we used MDA-MB-231 cells were performed in a similar manner. Monolayers of cells in 12-well plates were wounded with a P200 tip and subjected to imaging in the IncuCyte system. The TSctatch software was used to analyze the percentage of the field of view occupied by cells.

### Gelatin digestion assay

Coverslips were first treated with poly-d-lysine and glutaraldehyde, then coated with 0.1% pig gelatin solution containing Alexa Fluor 488–conjugated gelatin for 3 hours. The slides were quenched with 0.1% sodium borohydride solution prior to being seeded with 100,000 cells. Six hours after seeding, the cells were starved and treated with or without 200 pM TGFβ for 36 hours. The fixing, staining and imaging procedures are described above in the “Confocal microscopy” section.

### Statistical analysis

Unless otherwise mentioned, statistical analysis was done using an unpaired one-tailed Student’s *t*-test.

## Results

### BCAR3 expression correlates with favorable breast cancer disease outcome

**Figure 1:**
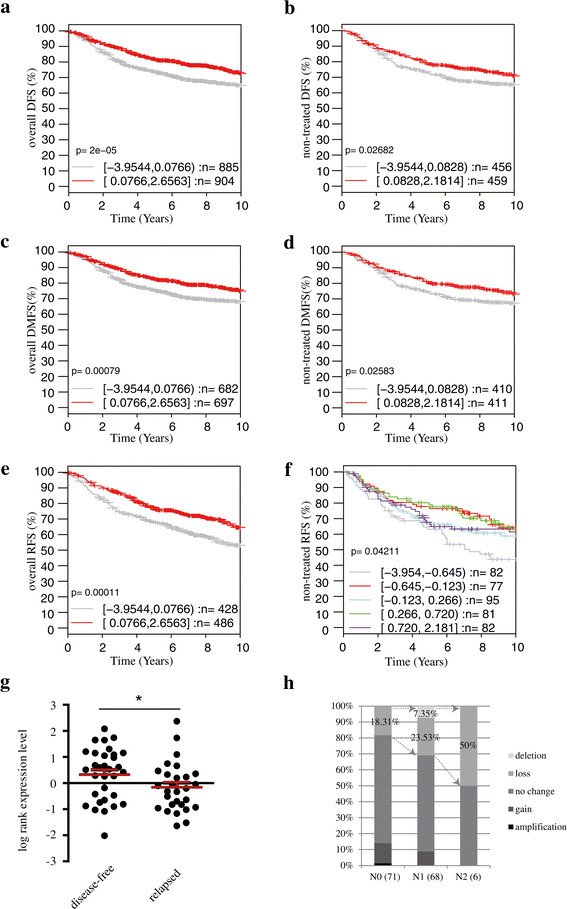
**Low BCAR3 expression predicts poor prognosis in human breast cancer. (a)** and **(b)** Kaplan-Meier survival curves generated by the GOBO gene expression–based outcome tool showing the status of disease-free survival (DFS) of a compiled cohort of breast cancer patients who underwent various treatment plans (a) and a compiled cohort of patients who received no systematic treatment (b). Patients were separated by the median signal intensity from an Affymetrix probe targeting BCAR3 (204032_at) in microarray analysis. Survival data of the high-expression group are shown by the red curve, and those of the low-expression group are shown by the gray curve. **(c)** and **(d)** Kaplan-Meier survival curves showing the status of distant metastasis-free survival (DMFS) of a compiled cohort of breast cancer patients who underwent various treatments (c) and a compiled cohort of patients who received no systematic treatment (d). Patients were separated by median signal intensity of 204032_at. **(e)** and **(f)** Kaplan-Meier survival curves showing status of relapse-free survival (RFS) of a compiled cohort of breast cancer patients who underwent various treatment plans (e) and a compiled cohort of patients who received no systematic treatment (f). Patients were separated by median signal intensity (e) or into five groups (f). **(g)** Dot plot of reading of the probe targeting BCAR3 extracted from the National Center for Biotechnology Information Gene Expression Omnibus dataset GDS807, which includes microarray readings of gene expression in microdissected primary tumors from patients who subsequently received tamoxifen treatment for 5 years. **(h)** Percentage loss of heterozygosity at BCAR3 alleles in breast tumors with increasing N stage.

BCAR3 is considered to be associated with aggressive disease phenotypes, as it promotes estrogen-independent cell proliferation, cell migration and contacts between cells and the extracellular matrix [5],[7],[9],[30],[31]. However, the results of a clinical study suggest that BCAR3 expression is a single factor that can predict favorable PFS of patients who receive tamoxifen treatment [10]. To investigate BCAR3’s clinical implications, we used GOBO [32] to generate Kaplan-Meier survival curves of breast cancer patients derived from published microarray datasets in the NCBI GEO database. We examined DFS (no relapse or distant metastasis) of two distinct cohorts of patients: a compiled cohort who underwent various treatments and a true prognostic cohort who received no systematic therapy. As shown in Figure 1a, in the compiled cohort, we found that those patients with low BCAR3 expression (gray) had significantly worse prognoses than patients with high BCAR3 levels (red), indicating that BCAR3 expression favors DFS for patients who received the various treatments. We also found a similar trend in the nontreated patient cohort (Figure 1b). This not only demonstrates a true prognostic value for BCAR3 but also implicates that loss of BCAR3 expression may be involved in breast cancer progression. We further analyzed and correlated BCAR3 expression levels with either distant metastasis or disease relapses. Consistently, we found BCAR3 expression to positively correlate with higher rates of DMFS in both an overall cohort and a nontreated cohort of patients (Figure 1c and 1d, respectively). Furthermore, BCAR3 expression also correlated with higher rates of RFS in the overall cohort (Figure 1e). In the nontreated cohort, when patients were separated by median expression level, no significant link was observed between BCAR3 and RFS (data not shown). However, when patients were separated into five groups based on the normalized readings of the probe corresponding to BCAR3 in the microarray experiments, we found that the group that expressed the lowest level of BCAR3 had significant worse prognosis, whereas the risk in the four other groups were comparable (Figure 1f). This suggests that a severe loss of BCAR3 expression correlates with markedly increased chance of tumor relapse. Notably, we also found similar correlations between low BCAR3 expression and poor prognosis of DMFS and RFS with the Breast Cancer Kaplan-Meier Plotter [33], which utilizes different but partially overlapping gene profiling datasets compared to those used in GOBO.

As ER+ and ER− breast tumors are distinct entities, we also stratified the analysis for ER+ and ER− tumors. We consistently observed similar correlations between low BCAR3 expression and poor outcomes in the ER+ cohort, and we did not observe statistically significant correlations in the ER− cohort (Additional file 1: Figure S1).

We further investigated whether established disease characteristics can be traced backward to BCAR3 expression levels in primary breast tumors. We analyzed both disease relapse and lymph node positivity, as they represent two critical indicators of the aggressiveness of the disease. To do so, we grouped patients based on established outcomes and surveyed for differences in BCAR3 expression between groups. In a cohort of patients with ER+ breast tumors treated with endocrine therapy for 5 years (NCBI GEO dataset GDS807) [34], those who developed disease relapse had lower levels of BCAR3 expression levels in their primary tumors as determined by log-transformed raw readings of the microarray dataset, which are expressed as relative fluorescence signaling intensities (Figure 1g). In particular, of the 32 patients with relatively high levels of BCAR3, 10 (31.25%) developed disease relapse. Of the 30 patients with relatively low levels of BCAR3, 19 (63.33%) developed disease relapse. These results, together with Kaplan-Meier analysis, highlight a high degree of overlap between patients with relatively low BCAR3 levels and patients with disease relapse in ER+ tumors. Additionally, using the ROCK breast cancer functional genomics database [35] from a CGH dataset designed to identify copy number abnormalities in breast cancer [36], we established a clear correlation between advanced tumor N stage/lymph node status and loss of heterozygosity at BCAR3 alleles in breast cancer patients. Indeed, combined loss and deletion of BCAR3 alleles increased from 18% in N0 tumors (no lymph node invasion), to 31% in N1 tumors (tumor cells in regional lymph nodes) and to 50% in N2 tumors (tumor cells in regional and distant lymph nodes). Altogether, these results indicate that loss of BCAR3 expression correlates with an invasive tumor phenotype with increased lymph node involvement. Our data also suggest that BCAR3 may play a favorable role in preventing disease progression in breast cancer patients.

### BCAR3 antagonizes TGFβ-induced Smad phosphorylation and accumulation of phospho-Smad3 in the nucleus

**Figure 2:**
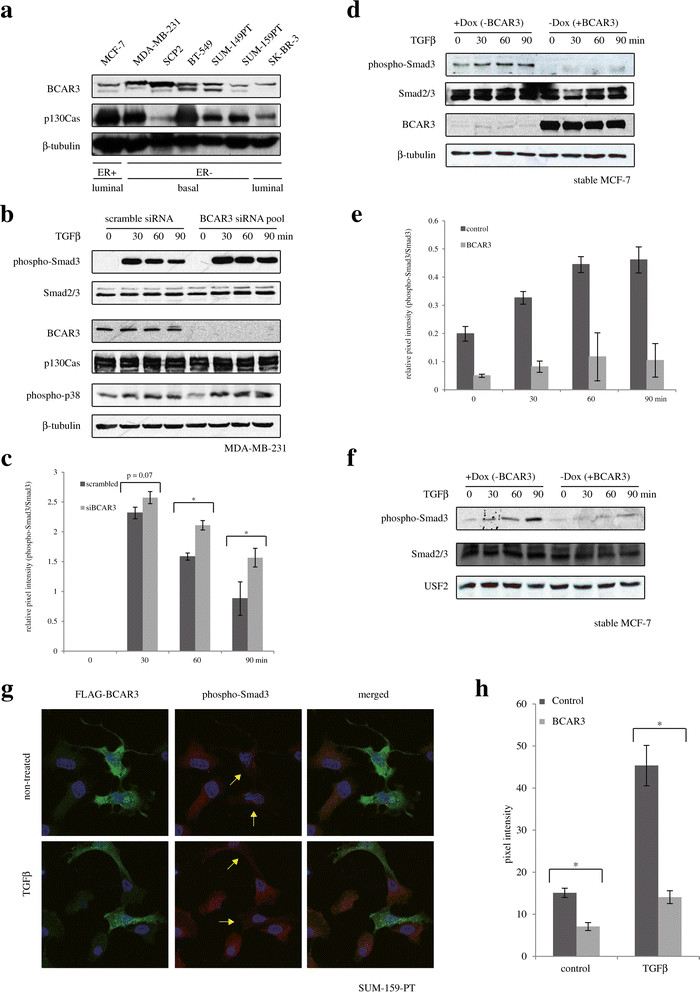
**BCAR3 antagonizes Smad activation. (a)** Total cell lysates from breast cancer cells were subjected to Western blot analysis for expression of BCAR3 and p130Cas. ER-, Estrogen receptor–negative; ER+, Estrogen receptor–positive. **(b)** MDA-MB-231 cells were transfected with a pool of two BCAR3 small interfering RNAs (siRNAs; 25 pM each), starved overnight and stimulated with 200 pM transforming growth factor β (TGFβ) 48 hours poststarvation for the indicated time periods. Levels of phospho-Smad3, total Smad2/3, BCAR3, p130Cas and phospho-p38 were examined by Western blotting. **(c)** Quantification of the relative signal density of phospho-Smad3 shown in b, normalized by signal density of Smad3. The results shown are from representative experiments (*n* = 3). *P < 0.05 by unpaired Student’s *t*-test. **(d)** Inducible MCF-7 cells were cultured with or without 1 μg/ml doxycycline (Dox), starved overnight and stimulated with 100 pM TGFβ 96 hours posttreatment for the indicted time periods. Levels of phospho-Smad3 and BCAR3 were examined by Western blot analysis (*n* = 2). **(e)** Quantification of relative signal density of phospho-Smad3 shown in d, normalized by signal density of Smad3. Results show a representative experiment (*n* = 2). **(f)** Levels of phospho-Smad3 in the nuclear extracts from the same pool of cells shown in (c) were examined by Western blot analysis (*n* = 2). **(g)** SUM-159-PT cells were seeded onto glass coverslips, transfected with FLAG-tagged AND-34 (mouse homologue of BCAR3) and starved overnight. Cells were then treated with or without 200 pM TGFβ for 1 hour and subjected to immunofluorescence microscopy and 4′,6-diamidino-2-phenylindole (DAPI) counterstaining. In the merged images, phospho-Smad3 is shown in red, FLAG-AND-34 is shown in green and cell nuclei are shown in blue (DAPI). Yellow arrows in the middle panels point at cells that express ectopic BCAR3. **(h)** Phospho-Smad3 signals in transfected and nontransfected cells were quantified from ten cells on at least five original LSM780 confocal microscopic images. Error bars show standard errors of the mean. An asterisk indicates a statistical difference between the two groups compared, as determined by unpaired Student’s *t*-test (*P < 0.05).

We then sought to investigate the molecular mechanisms by which BCAR3 exerts this potentially protective role. Interestingly, a BCAR3-interacting protein, p130Cas, was previously shown to directly interact with Smad2/3, thereby blocking Smad C-terminal serine phosphorylation and activation, resulting in an inhibition of TGFβ signaling [37],[38]. As the TGFβ/Smad signaling pathway plays a prominent role in breast cancer progression and tumor metastasis, we investigated whether BCAR3 could regulate TGFβ/Smad signal transduction. We initially examined the relative protein expression levels of BCAR3 and p130Cas in a panel of breast cancer cell lines representing different molecular subtypes and phenotypes of breast tumors. We found that, similarly to previously reported findings [39], BCAR3 expression levels were relatively high in estrogen-independent breast cancer cells (Figure 2a and Additional file 2: Figure S2). Additionally, BCAR3 expression generally correlated with breast cancer subtype. Luminal-like MCF-7 and SK-BR-3 cells express relatively low levels of BCAR3, whereas basal-like MDA-MB-231, SCP2, BT-549 and SUM-149PT cells expressed relatively high levels of BCAR3 (Figure 2a). However, SUM-159PT cells, which are ER- and estrogen-independent in culture and as xenografts, also expressed a relatively low level of BCAR3 (Figure 2a). This may likely be due to the anaplastic nature of the origin of these cells [40]. In addition, BCAR3 immunoblotting revealed two bands of close molecular weights. Both bands appeared to be BCAR3-specific, as shown by the BCAR3 siRNA knockdown (Figure 2b). The presence of two bands indicates that BCAR3 likely undergoes posttranslational modification. Furthermore, p130Cas is fairly abundant in all cells tested, and its expression did not seem to correlate with either ER status or cancer subtype (Figure 2a). These results suggest that, though p130Cas expression may be a universal event in most types of breast cancer cells and tumors, high BCAR3 expression is likely specific in cells of a basal-like breast cancer phenotype.

These results also allowed us to choose ideal cell line models for loss-of-function and gain-of-function approaches to investigate whether BCAR3 is involved in regulating the TGFβ/Smad signaling axis. For loss-of-function assays, we transfected siRNAs targeting BCAR3 into MDA-MB-231 cells or into its single-cell progeny SCP2. SCP2 cells are a subprogeny of MDA-MB-231 cells that specifically metastasize to the bone [26],[41]. These cell lines expressed relatively high levels of endogenous BCAR3. For gain-of-function assays, we expressed ectopic BCAR3 in either MCF-7 cells or SUM-159-PT cells. These cell lines expressed relatively low levels of endogenous BCAR3. MCF-7 and SUM-159 cells appeared to have relatively stronger phospho-Smad3 levels than MDA-MB-231 cells and SCP2 cells, as determined by Western blot analysis (Additional file 2: Figure S2).

In MDA-MB-231 cells, TGFβ induced Smad3 phosphorylation in time-dependent manner. Interestingly, this effect was potentiated and prolonged when BCAR3 gene expression was silenced using a pool of two siRNAs targeting BCAR3 (Figure 2b and 2c). Similar results were observed in SCP breast cancer cells (Additional file 3: Figure S3). In addition, we did not observe remarkable effects of BCAR3 siRNA on p130Cas protein levels or TGFβ-induced p38 phosphorylation (Figure 2b). These results suggest that endogenous BCAR3 inhibits Smad signaling.

To further address this inhibitory role, we examined the effect of ectopic BCAR3 on Smad signaling in MCF7 cells. MCF-7 cells express a low endogenous BCAR3 level, as determined by Western blot analysis, and they represent an ideal model for overexpression studies. We used an inducible, stable MCF-7 cell line that overexpresses BCAR3 under the control of a TeT-off promoter under normal culture conditions (generated by Dr. Amy Bouton, Microbiology, Immunology, and Cancer Biology, University of Virginia School of Medicine, Charlottesville, VA, USA) [7]. As shown in Figure 2d, removing doxycycline greatly induced BCAR3 expression in these cells (middle panel). TGFβ-induced Smad phosphorylation, observed in the absence of BCAR3, was remarkably blocked when BCAR3 was overexpressed (Figure 2d and 2e). As activated Smad translocated into the cell nucleus, we analyzed both nuclear total Smad2/3 and phospho-Smad3 levels of MCF7 cells, treated or not with doxycycline. Removing doxycycline in the culture medium resulted in decreases in accumulation of phospho-Smad3 in the cell nucleus in response to TGFβ (Figure 2f). Together, these results demonstrate that BCAR3 expression inhibits TGFβ-induced Smad phosphorylation.

The MCF-7 cells that we used are derived from a stable, inducible clone of cells. To rule out the possibility of clone-specific effects, we took another approach to examine the role of ectopic BCAR3 on Smad activation. We transiently transfected FLAG-tagged AND-34 (the mouse homologue of BCAR3) into SUM-159PT, which is another breast cancer cell line that expresses low levels of endogenous BCAR3. Because of low transfection efficiency, we studied the effects of ectopic BCAR3 on Smad signaling by confocal microscopy imaging rather than by Western blotting. This also allowed us to observe both cells overexpressing BCAR3 and nonoverexpressing cells in the same field. Interestingly, cells expressing FLAG-tagged AND-34/BCAR3 displayed weaker overall phospho-Smad3 signals, compared to nontransfected cells in the same field, under both resting and TGFβ-stimulated conditions (Figure 2g, yellow arrows). We further quantified Smad3 phosphorylation levels of ten cells on at least five original confocal images for each experimental condition. In cells transiently transfected with BCAR3, signaling of TGFβ-induced phospho-Smad3 decreased to about 40% of that in nontransfected cells (Figure 2h). Taken together, our results demonstrate that BCAR3 antagonizes TGFβ-induced Smad activation in several breast cancer cell lines.

### BCAR3 inhibits TGFβ-mediated Smad transcriptional activity and target gene expression

**Figure 3:**
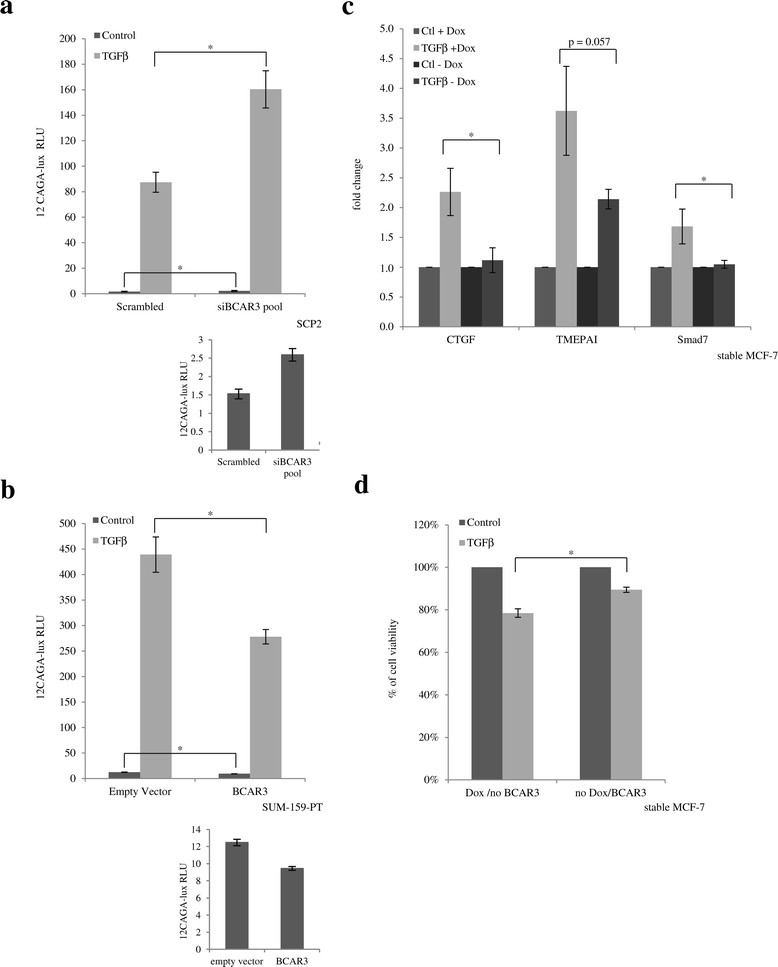
**BCAR3 antagonizes function of canonical transforming growth factor β signaling. (a)** SCP2 cells were cotransfected with a (CAGA)12-lux luciferase reporter construct, constitutive β-galactosidase construct and 50 pM scrambled small interfering RNA (siRNA) control or a pool of siRNAs targeting BCAR3 (25 pM each). Cells were starved overnight, stimulated with or without 100 pM transforming growth factor β (TGFβ) and subjected to luciferase assays. Luciferase activities were normalized by β-galactosidase activity and are represented as relative luciferase units (RLUs). Error bars show standard errors of the mean of three independent experiments. An asterisk indicates a statistically significant difference as determined by unpaired Student’s *t*-test (*P < 0.05). The inset under the figure shows basal luciferase activities. **(b)** SUM-159-PT cells were cotransfected with (CAGA)12-lux luciferase reporter construct, constitutive β-galactosidase construct and FLAG-tagged AND-34. Luciferase assays were performed as described in **(a)**. The inset under the figure shows basal luciferase activities. **(c)** Inducible MCF-7 cells were cultured with or without doxycycline (Dox) for 72 hours, starved overnight and stimulated with 100 pM TGFβ for 24 hours. mRNA levels of three Smad-dependent genes (connective tissue growth factor (CTGF), transmembrane prostate androgen induced RNA (TMEPAI) and Smad7) were examined by real-time PCR. Bars show fold changes of induction by TGFβ. Error bars show SEM of three independent experiments. An asterisk indicates a statistically significant difference as determined by unpaired Student’s *t*-test (*P < 0.05). **(d)** Inducible MCF-7 cells were cultured with or without Dox for 72 hours, starved overnight and stimulated with 100 pM TGFβ for 72 hours. Cell viability was examined by using a 3-(4,5-dimethylthiazol-2-yl)-2,5-diphenyltetrazolium bromide (MTT; thiazolyl blue tetrazolium bromide) assay. Error bars show SEM of three independent experiments. An asterisk indicates statistically significant difference (*P < 0.05).

We next investigated whether modulating BCAR3 levels could alter Smad-mediated transcriptional activity using a Smad-responsive reporter construct, (CAGA)12-lux, which contains 12 repeats of minimal Smad binding site upstream of the firefly luciferase open reading frame. As shown in Figure 3a, TGFβ strongly induced luciferase activity in SCP2 cells transfected with the (CAGA)12-lux construct. However, knocking down endogenous BCAR3 using a pool of two specific siRNAs against BCAR3 resulted in significant increases in both basal and TGFβ-induced luciferase activity (Figure 3a). Consistently, transient transfection of AND-34 (mouse homologue of BCAR3) into SUM-159-PT cells resulted in significant decreases in both basal and TGFβ-induced luciferase activity (Figure 3b). We further tested, by real-time PCR, whether BCAR3 could alter the expression of *bona fide* TGFβ target genes. *CTGF* [42], *TMEPAI* [43] and *Smad7* [44] were previously reported to be upregulated by TGFβ in a Smad-dependent manner. Ectopic BCAR3 expression in MCF-7 cells almost completely blocked TGFβ’s ability to induce CTGF and Smad7 and also remarkably impaired TGFβ’s ability to induce TMEPAI expression (Figure 3c). The difference in effectiveness may be due to involvement of Smad-independent mechanisms downstream of TGFβ. Taken together, these results indicate BCAR3 antagonizes Smad transcriptional activity.

MCF-7 cells are luminal-like, estrogen-responsive and relatively well-differentiated. These cells retain a partial cytostatic response to TGFβ. We therefore investigated whether ectopic BCAR3 could antagonize TGFβ’s growth-inhibitory effects in these cells. Stable MCF-7 cells cultured with doxycycline expressed low levels of BCAR3. TGFβ treatment resulted in 25% reduction in cell viability as determined by an MTT cell viability assay. This is consistent with the results of similar experiments carried out by others [45],[46]. Stable BCAR3 expression, on the other hand, reversed TGFβ’s effect, resulting in less than 10% reduction in cell viability (Figure 3d).

Growth curves were created for cells cultured with or without doxycycline and treated with or without 200 pM TGFβ. TGFβ led to about a 20% decrease in cell confluence at 84 hours under both conditions. TGFβ stop decreases in cell confluence in cells cultured without doxycycline (BCAR3 overexpression), but continue to decrease cell confluence in cells cultured with doxycycline (data not shown).

### BCAR3 antagonizes TGFβ promigratory and proinvasive responses

A hallmark effect of TGFβ in breast cancer cells, particularly in basal-like and triple-negative cells, is single-cell migration [17],[26],[28],[47]. Lines of evidence suggest that TGFβ reprograms transcriptional profiles in breast cancer cells to induce epithelial-to-mesenchymal transition, formation of filopodia and switching from collective cell migration to single-cell migration and ultimately to facilitate intravasation [26],[47]-[49]. These effects, although not necessarily concomitant, highlight the promigratory role of TGFβ. As such, we investigated whether modulating BCAR3 levels in basal-like breast cancer cells could affect TGFβ-induced cell migration. For this purpose, we silenced endogenous *BCAR3* gene in SCP2 cells using two specific siRNAs, and examined TGFβ-induced cell migration using the IncuCyte time-lapse video imaging migration assay as previously described [26]. This method couples a wound-healing assay with quantitative imaging and presents cell migration by relative wound density, which is the real-time ratio between cell densities within the initial wound area to cell density of the adjacent nonwounded area. As such, this method precludes a net change in cell number over time due to cell proliferation.

**Figure 4:**
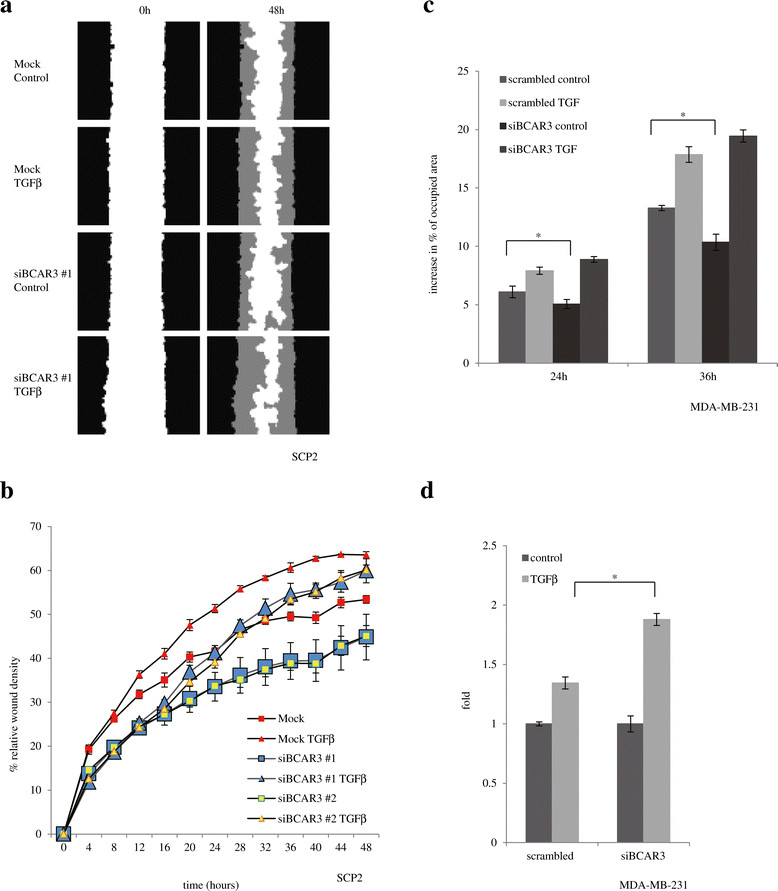
**Knocking down BCAR3 promotes transforming growth factor β**–**induced cell migration. (a)** SCP2 cells were transfected, scratched and treated as described in the Methods section and subjected to real-time migration assays. The images are computer-generated pictures showing positions of cell boundaries at the beginning of the assay (time 0) and at the end of the assay (48 hours). The black regions represent occupancy of cells after initial scratching, the white regions represent empty space not filled by cells (the scratch) and the gray regions represent initial scratch areas filled by cells at the end of the assay (migration). The images are representative of three independent experiments. Because of space limitations, the images for small interfering RNA (siRNA) #2 are not shown. **(b)** Migration profiles over time were compared for mock-transfected cells and cells transfected with two different BCAR3 siRNAs. Error bars show standard error of the mean of six biological replicates in one experiment. **(c)** and **(d)** MDA-MB-231 cells were transfected, scratched using a P200 tip and stimulated with or without 200 pM transforming growth factor β (TGFβ). Cells were subjected to real-time cell migration assays using the IncuCyte automated imaging system. Images obtained at 0, 24 and 36 hours were analyzed using TScratch to automatically recognize wound area and calculate the percentage of the fields of view occupied by cells. The results show cell migration expressed as increases in area percentage at 24 hours and 36 hours (c) and fold induction by TGFβ at 36 hours (d), respectively. The percentages of the fields of view were quantified from ten image sets of a representative experiment (*n* = 3). Error bars show standard error of the mean. An asterisk indicates statistical significance (*P < 0.05) as determined by unpaired Student’s *t*-test.

TGFβ treatment induced time-dependent migration of SCP2 cells (Figure 4a and 4b). The effect was detectable as early as 12 hours following stimulation of the cells and increased further over time to reach a plateau at 48 hours. Cell density in the wounded area was about 52% of that of the adjacent area. TGFβ treatment resulted in about a 10% increase in relative cell density, indicative of more cells in the wounded area. Individual siRNAs against BCAR3 decreased cell migration, marked by only about a 42% increase in relative wound density after 48 hours (Figure 4b). This is consistent with previous findings that ectopic BCAR3 expression increases cell migration [9]. Interestingly, cells transfected with the siRNAs displayed an increased response to TGFβ, marked by about a 15% increase in relative cell density (Figure 4b). The two siRNA constructs seemed to have similar effects.

We further transfected a pool of the two BCAR3 siRNAs into MDA-MB-231 cells and studied cell migration, measured by percentage of the image area occupied by cells. We selected the images that were comparable in initial wound areas (40% to 43% of the image occupied by wounding) and measured increases in areas occupied by cells at 24 and 36 hours. Similar to the above-mentioned results, we observed that MDA-MB-231 transfected with BCAR3 siRNA migrated slower. At 36 hours, cells transfected with scrambled siRNA migrated into 13% of the total area, whereas cells transfected with BCAR3 siRNA migrated into about 10% of the total area (Figure 4c). However, TGFβ induced more cell migration in cells transfected with BCAR3 siRNA than in cells transfected with scrambled siRNA, as determined by directly measuring the areas (9% vs. 3.5% at 36 hours; Figure 4c) or by fold change (1.9-fold vs. 1.35-fold at 36 hours; Figure 4d). As such, these data indicate that endogenous BCAR3 is an antagonistic molecule of TGFβ-induced cell migration.

**Figure 5:**
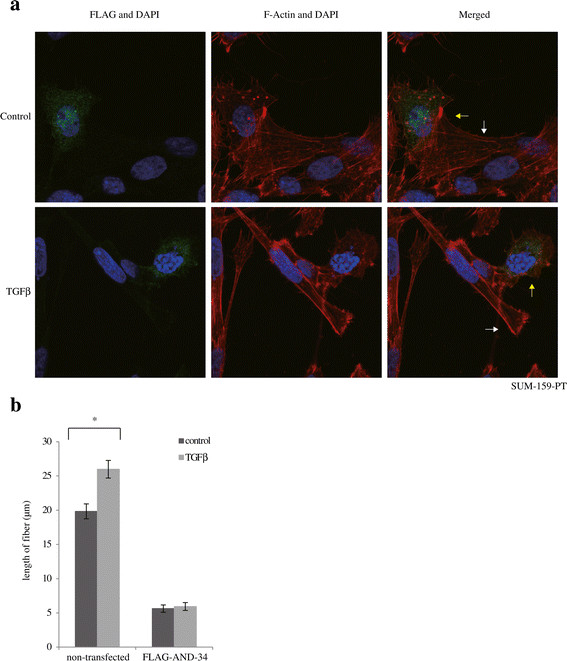
**Overexpressing BCAR3 antagonizes transforming growth factor β**–**induced cytoskeleton rearrangement. (a)** SUM-159-PT cells were transfected with FLAG-tagged AND-34, starved and treated with or without 200 pM transforming growth factor β (TGFβ) for 24 hours, then subjected to immunofluorescence microscopy. In the merged images, FLAG-tagged AND-34 is shown in green, F-actin is shown in red and cell nuclei are shown in blue (4′,6-diamidino-2-phenylindole (DAPI)). The results are representative of two independent experiments. **(b)** The lengths of stress fibers are quantified from five cells in pairs per condition on different LSM780 confocal microscopic images. Error bars show standard error of the mean. An asterisk indicates statistical significance (*P < 0.05) as determined by unpaired Student’s *t*-test.

We further overexpressed FLAG-tagged AND34 in SUM-159 cells and examined whether it antagonized TGFβ’s promigratory effects by confocal microscopy. Single-cell migration requires organization of actin into treadmilling filaments oriented toward lamellipodia [50]. In the absence of FLAG-tagged AND-34 overexpression, 24 hours of TGFβ treatment induced a network of elongated actin stress fibers aligned toward filopodia-like structures, indicative a promigratory phenotype (Figure 5a, white arrow). In cells transfected with FLAG-tagged AND34, fluorescence signaling corresponding to the FLAG tag locates predominantly in the cytoplasm and also overlaps with flagellum-like structures on cell membranes. Transfected cells, although they still contained actin filaments, failed to display dominant filopodia-like structures. Rather, they contained relatively short, branched fibers that oriented in all directions, even when they were stimulated with TGFβ (Figure 5a, yellow arrow). We observed these phenotypes with virtually all transfected cells. The length of stress fibers was quantified in the presence or absence of overexpressed FLAG-tagged BCAR3. The results indicate that BCAR3 overexpression leads to shorter stress fibers and interferes with the TGFβ effect on fiber elongation (Figure 5b). Taken together, these data demonstrate that BCAR3 could antagonize TGFβ’s promigratory function, likely by interfering with TGFβ-mediated actin filament rearrangement and filopodia formation.

**Figure 6:**
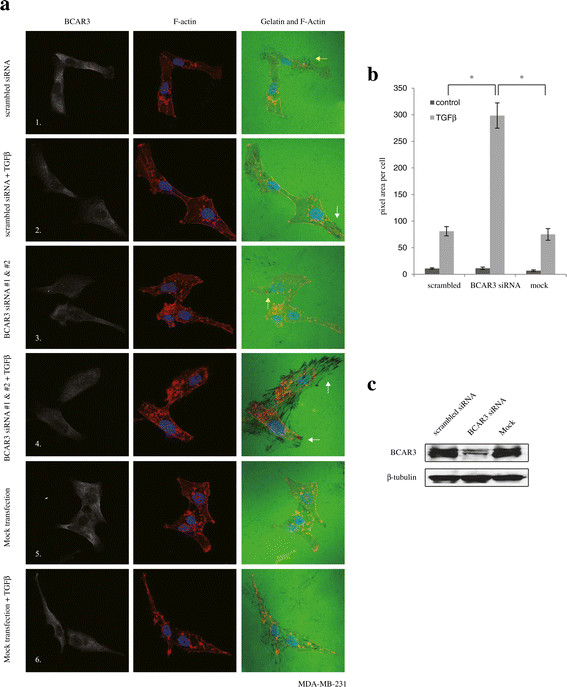
**Knocking down BCAR3 promotes transforming growth factor β**–**induced gelatin matrix digestion. (a)** SCP2 cells were transfected either with 50 pM scrambled control siRNA (panels 1 and 2), or with 50 pM BCAR3 siRNA (panels 3 and 4), or they were mock-transfected (panels 5 and 6) for 48 hours. Cells were then seeded onto coverslips coated with Alexa Fluor 488–conjugated gelatin, treated with or without 200 pM transforming growth factor β (TGFβ) and allowed to digest the matrix for 36 hours. Representative images show immunofluorescence staining of BCAR3 (white), actin filaments (red) and gelatin matrix (green). The results represent two independent experiments. **(b)** The total areas of digestion (pixel count) were quantified for ten cells in each condition in seven to ten LSM780 confocal microscopic image files from the two experiments. Error bars show SEM, and an asterisk indicates statistical significance (*P < 0.05) determined by unpaired Student’s *t*-test. **(c)** Western blot showing the effect of BCAR3 siRNA using total cell lysates of the same pool of cells used for the gelatin degradation assay.

Digestion of extracellular matrix is both a major event during cell invasion and an indication of aggressive properties of cancer cells. TGFβ acts as a potent proinvasive factor in breast cancer cells. It has been reported that, in a matrix degradation assay, TGFβ increased digestion of gelatin matrix by MDA-MB-231 cells [51]. As such, we used this well-established assay to examine whether ectopic BCAR3 expression could antagonize TGFβ’s effects on matrix degradation by MDA-MB-231 cells. As shown in Figure 6a, mock-transfected cells (row 1) cells transfected with scrambled siRNA (row 5) displayed matrix digestion ability under nonstimulated conditions. Indeed, when plated on coverslips coated with Alexa Fluor 488–tagged gelatin, these cells produced small, scattered areas of digestion underneath their bodies, observed as dark spots under a confocal microscope (Figure 6a, row 1, yellow arrow). Mock-transfected and control siRNA-transfected MDA-MB-231 cells, when treated with TGFβ, displayed a clear increase in the total area of digested matrix (Figure 6a, rows 2 and 6, and Figure 6b). Many of these digested areas were elongated, indicative of cell movement during matrix digestion. Noticeably, instead of scattering underneath the cell body, elongated digestion spots tended to aggregate at the cell protrusions, overlapping with the lamellipodia-like structures formed by bundled actin filaments (Figure 6a, row 2, white arrow). These data suggest that, in addition to increasing the digestion of gelatin matrix, TGFβ also affects the localization of the invadopodia in MDA-MB-231 cells and remodels their structure from a scattered pattern to an aggregated pattern. Interestingly, transfection of MDA-MB-231 cells with a pool of two BCAR3 siRNAs significantly potentiated TGFβ’s effects on matrix digestion, as illustrated by the large areas of digested gelatin (Figure 6a, row 4, white arrows). Particularly, multiple elongated spots formed in these areas, which are roughly parallel to each other. These spots also appeared to be longer than those formed in control cells. We verified the efficiency of the siRNA knockdown from the same pool of cells (Figure 6c). Taken together, these results indicate that BCAR3 gene silencing, by means of RNA interference, potentiates TGFβ-induced invadopodia activity and matrix digestion, suggesting that endogenous BCAR3 inhibits TGFβ-induced invadopodia remodeling and matrix digestion.

### BCAR3 requires p130Cas to antagonize Smad signaling

**Figure 7:**
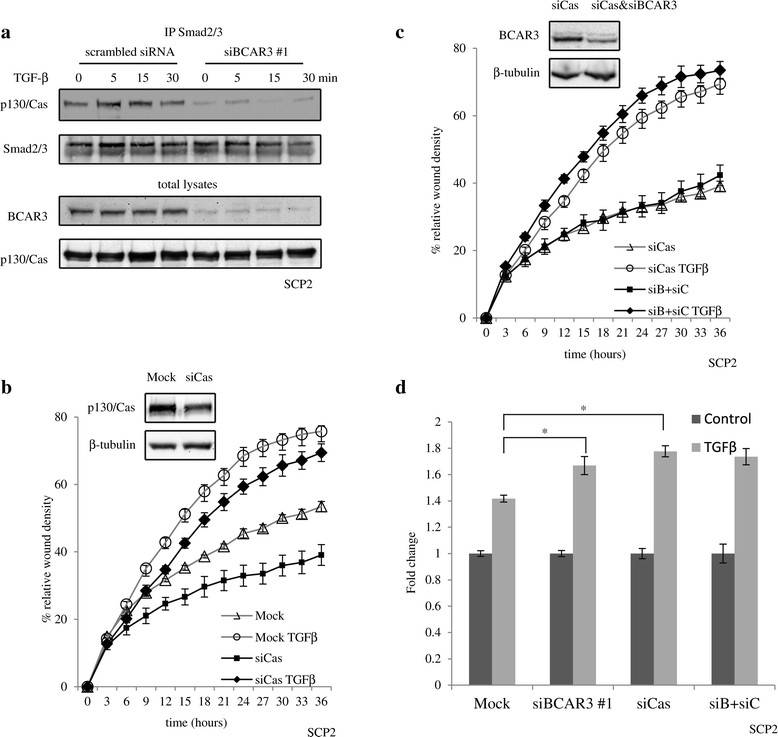
**BCAR3 requires p130Cas to antagonize transforming growth factor β signaling. (a)** SCP2 cells were transfected with BCAR3 small interfering RNA (siRNA), starved overnight and stimulated with 200 pM transforming growth factor β (TGFβ). Smad2/3 was immunoprecipitated (IP), and levels of p130Cas in the precipitant were examined by Western blot analysis. **(b)** and **(c)** SCP2 cells were transfected with BCAR3 siRNA or p130Cas siRNA or together, then starved, stimulated with 200 pM TGFβ and subjected to IncuCyte cell migration assays. Real-time migration profiles were compared for mock-transfected cells and cells transfected with p130Cas siRNA (b) and for cells transfected with p130Cas siRNA and both siRNAs (c). Error bars show standard error of the mean for six biological replicates of a representative experiment (*n* = 3). **(d)** The effects of TGFβ on cell migration at the endpoint (36 hours) are plotted as fold changes of relative wound density. Error bars show standard error of the mean. An asterisk indicates statistical significance (*P < 0.05) as determined by unpaired Student’s *t*-test.

Previous studies indicated that the p130Cas physically interacts with Smad2/3 and antagonize Smad activation [37],[38]. As p130Cas also interacts with BCAR3, this prompted us to investigate whether p130Cas is involved in BCAR3-mediated inhibition of TGFβ/Smad signaling. We precipitated Smad2/3 using a rabbit polyclonal antibody (FL-425, Santa Cruz Biotechnology) from total cell lysates of SCP2 cells, and found p130Cas to be constitutively associated with Smad2/3 (Figure 7a). However, knocking down endogenous BCAR3 expression impaired this association, suggesting that endogenous BCAR3 promotes the interaction between p130Cas and Smad2/3 (Figure 7a). Membranes were reprobed with the anti-BCAR3 antibody, but no association could be detected between Smads and BCAR3 (data not shown).

We next investigated whether BCAR3 also required p130Cas to modulate TGFβ-induced cell migration. Transfection of SCP2 cells with siRNAs targeting either BCAR3 or p130Cas both decreased basal cell migration and increased TGFβ-induced cell migration (Figure 4b and Figure 7b, respectively). However, upon silencing of p130Cas, BCAR3 siRNA lost the ability to further potentiate TGFβ-induced cell migration (Figure 7c). Indeed, TGFβ stimulation resulted in about a 40% increase in cell migration in mock-transfected cells at 36 hours poststimulation. Transfection of cells with BCAR3 siRNA, or p130Cas siRNA, or both, resulted in about 70% increases. Cotransfection of the two siRNAs did not have a more than additive effect (Figure 7d), suggesting that BCAR3 likely requires the presence of p130Cas to antagonize TGFβ function. Altogether, these data suggest that BCAR3 modulates an interaction between p130Cas and Smad2/3, thereby blocking TGFβ/Smad-mediated cell migration.

### BCAR3 mediates a positive feedback of TGFβ signaling in breast cancer cells

**Figure 8:**
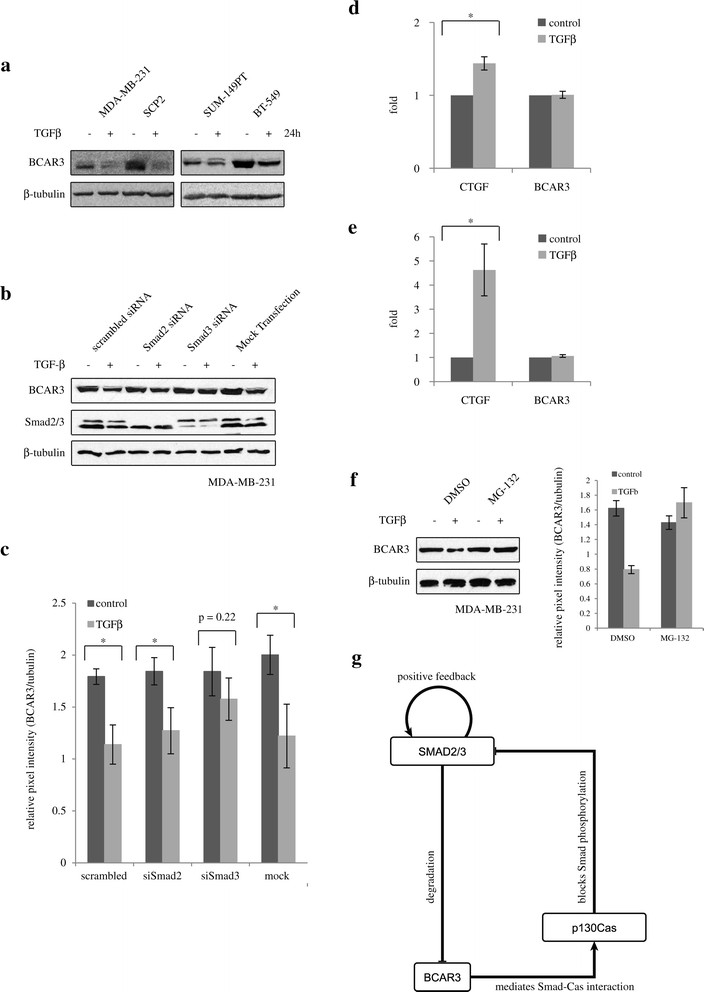
**Transforming growth factor β downregulates BCAR3 in a Smad-dependent manner. (a)** Basal-like breast cancer cells were starved and treated with 200 pM transforming growth factor β (TGFβ) for 24 hours. Levels of BCAR3 protein expression were examined by Western blot analysis. **(b)** MDA-MB-231 cells were transfected with Smad2 small interfering RNA (siRNA) or Smad3 siRNA for 48 hours, then starved and treated with 200 pM TGFβ for 24 hours. Levels of BCAR3 protein expression were examined by Western blot analysis. **(c)** The signal intensity of BCAR3 was quantified and normalized to that of β-tubulin. The results show normalized signal intensity calculated from three independent experiments, and error bars show standard error of the mean. An asterisk indicates statistical significance (*P* < 0.05) as determined by unpaired Student’s *t*-test. **(d)** and **(e)** MDA-MB-231 (d) or BT-549 (e) cells were treated with or without 100 pM TGFβ for 24 hours. Total RNA was extracted, reverse-transcribed and subjected to real-time PCR to examine expression of connective tissue growth factor (CTGF) and BCAR3. The results show average fold changes calculated from three independent experiments, and error bars show standard error of the mean. An asterisk indicates statistical significance (*P < 0.05) as determined by unpaired Student’s *t*-test. **(f)** MDA-MB-231 cells were starved and treated with 10 μM MG-132 or DMSO as vehicle control for 1 hour, then stimulated with or without 200 pM TGFβ for 24 hours, as indicated. BCAR3 protein levels were examined by Western blot analysis (left panel). Relative BCAR3 protein levels, compared to β-tubulin, were quantified by densitometry for two separate experiments. DMSO, Dimethyl sulfoxide. **(g)** Model of BCAR3 in mediating a positive feedback loop downstream of the TGFβ/Smad signaling axis.

Extensive studies of the molecular functions of BCAR3 have been conducted; however, there has been no report published to date on how BCAR3 gene expression is regulated. As cellular signaling pathways are often modulated by feedback regulatory loop mechanisms to ensure defined signaling intensity and duration, we investigated whether TGFβ signaling itself could modulate BCAR3 gene expression. For this purpose, we stimulated a panel of basal-like breast cancer cell lines with TGFβ for 24 hours and examined the protein levels of BCAR3. As shown in Figure 8a, TGFβ treatment resulted in a remarkable decrease in BCAR3 protein levels in all cell lines tested, highlighting BCAR3 as a novel target for TGFβ signaling. Furthermore, our results indicate that TGFβ-mediated suppression of BCAR3 gene expression depends on the canonical Smad signaling, as knocking down Smad3, but not Smad2, in MDA-MB-231 cells partially blocked TGFβ’s effect in decreasing BCAR3 protein expression (Figure 8b). Quantification of the data was performed in three separate experiments, and statistical analysis was done using unpaired Student’s *t*-tests (Figure 8c). We also observed similar results in BT-549 cells (data not shown). The ability of TGFβ to decrease BCAR3 protein levels is likely not through transcriptional regulation. We did not observe statistically significant decreases in BCAR3 mRNA levels following TGFβ treatment in MDA-MB-231 cells and BT-549 cells (Figure 8d and 8e, respectively). TGFβ’s effect of decreasing BCAR3 protein levels may involve the proteasome pathway, as treating MDA-MB-231 cells with MG-132 (for 1 hour), a proteasome inhibitor, abolished this effect (Figure 8f). Taken together, these results suggest a positive feedback loop mechanism by which TGFβ/Smad signaling represses expression of its own inhibitory molecule, BCAR3, further leading to enhanced TGFβ/Smad signaling in breast cancer cells (Figure 8g).

## Discussion

In this study, we defined a novel role for BCAR3 in which it antagonizes the canonical TGFβ signaling by promoting an interaction between p130Cas and the Smad transcription factors. The inhibitory effect of BCAR3 on TGFβ/Smad signaling was observed in all breast cancer cell lines tested, regardless of their molecular phenotype and biological response to TGFβ. Although TGFβ efficiently induces growth arrest in normal mammary epithelial cells and cells of early breast carcinoma, these cytostatic responses are lost in more advanced, invasive breast tumors and replaced by TGFβ-induced tumor-promoting and prometastatic responses [19],[52]. In invasive, basal-like breast cancer cells, such as MDA-MB-231, SCP2 and SUM-159-PT, BCAR3 antagonizes several of TGFβ’s proinvasive effects, such as cell migration, formation of filopodia-like structures and digestion of gelatin matrix. However, in the luminal-like, ER+ MCF7 cells, BCAR3 overexpression antagonized TGFβ’s ability to decrease cell viability. These findings imply that the novel function of BCAR3 of Smad signaling inhibition is likely a conserved mechanism among the different molecular subtypes of breast cancer.

It is important to mention that BCAR3’s roles in aggressive behavior of breast cancer cells are twofold. On one the hand, BCAR3 activates several small GTPases that play key roles in cell migration, including Rac1 and CDC42 [31],[39]. In fact, the results of a previous study [53] and our present results both demonstrate that knocking down BCAR3 in basal-like breast cancer cells impairs cell migration. On the other hand, our results show that BCAR3 antagonizes TGFβ-mediated cell migration and invasion. These results do not necessarily contradict each other, as one demonstrates BCAR3’s role in regulating cell migration itself and the other demonstrates BCAR3’s involvement in regulating TGFβ signaling and TGFβ’s biological effects. Follow-up studies need to be done to address the effects of BCAR3 on cell migration, invasion and metastasis in a physiological context, such as a xenograft experimental metastasis model.

In addition, we found that TGFβ decreased BCAR3 protein expression in multiple breast cancer cell lines. This effect appeared to be Smad3-dependent and proteasome-dependent. These data are the first to reveal a molecular mechanism that regulates BCAR3 expression. More importantly, our data define a positive feedback mechanism downstream of TGFβ/Smad signaling. It is known that a few negative feedback loops exist that fine-tune TGFβ signaling, such as those mediated by Smad7 [44] and GRK2 [54]. Both of these targets have been shown to be upregulated by TGFβ, further leading to termination of Smad signaling [44],[54],[55]. To our knowledge, BCAR3 serves as the first example of a positive feedback loop of TGFβ/Smad signaling, whereby TGFβ signaling itself can decrease the expression levels of its inhibitor BCAR3, further leading to enhanced TGFβ/Smad signaling. Such a mechanism may be important for maintaining a steady response to TGFβ.

It has been established that active Smad signaling contributes to breast cancer local invasion and distant metastasis. Having defined BCAR3 as a novel TGFβ/Smad inhibitory molecule, this may account for the relatively low BCAR3 expression levels observed in primary breast tumors associated with worse prognosis and higher levels of disease progression. Indeed, low BCAR3 levels in tumor cells may lead to potent responses to TGFβ. The results of our clinical data survey and analysis define BCAR3 as a single factor whose expression level is predictive of clinical outcomes in breast cancer patients. Moreover, we also found that patients with lymph node metastasis tend to carry loss of heterozygosity at BCAR3 alleles, indicating that BCAR3 likely plays a role in preventing disease progression.

## Conclusions

Taken together, the results of our study identify a novel positive feedback loop mechanism downstream of the canonical TGFβ/Smad signaling axis, mediated by a breast cancer anti-estrogen resistance gene, *BCAR3*. We report a novel role of BCAR3 to antagonize Smad signaling, efficiently leading to inhibition of the TGFβ’s biological functions in breast cancer cells. Our results also highlight potential prognostic value of BCAR3 in human breast cancer, as we found low BCAR3 expression levels in primary breast tumors to be correlated with poor outcomes, regardless of treatment plans. Our study provides new insights into BCAR3’s mechanism of action and suggests a need to reevaluate the implications of BCAR3’s role in breast cancer pathology.

## Additional files

## Electronic supplementary material

Additional file 1: Figure S1.: Low BCAR3 expression predicts poor prognosis in ER+ breast cancer. **(a)** and **(b)** Kaplan-Meier survival curved generated by GOBO gene expression-based outcome tool, showing status of disease-free survival (DFS) of ER+ breast cancer patients (a) and ER− patients (b). Patients were separated by median of signal intensity from an Affymetrix probe targeting BCAR3 (204032_at) in microarray analysis. Survival data of the high-expression group are shown by the red curve, and those of the low-expression group are shown by the gray curve. **(c)** and **(d)** Kaplan-Meier survival curves showing status of distant metastasis-free survival (DMFS) of ER+ patients (c) and ER− patients (d). Patients were separated by median of signal intensity from 204032_at. **(e)** and **(f)** Kaplan-Meier survival curves showing status of relapse-free survival (RFS) of ER+ patients (e) and ER− patients (f). (PDF 458 KB)

Additional file 2: Figure S2.: Protein levels of phospho-Smad3 and Smad2/3 in selected breast cancer cells. Levels of phospho-Smad3, Smad2/3, BCAR3 and p130Cas in total cell lysates of MCF-7, SUM-159PT, SCP2 and MDA-MB-231 cells were determined by Western blot analysis. (PDF 469 KB)

Additional file 3: Figure S3.: Knocking down BCAR3 enhances TGFβ-induced Smad3 phosphorylation in SCP2 cells. SCP2 cells were transfected with BCAR3 siRNA ((#1: SASI_Hs01_00236261), starved overnight and stimulated with 200 pM TGFβ 48 hours poststarvation for the indicated time periods. Levels of phospho-Smad3 and BCAR3 were examined by Western blot analysis. (PDF 446 KB)

Below are the links to the authors’ original submitted files for images.

Authors’ original file for figure 1

Authors’ original file for figure 2

Authors’ original file for figure 3

Authors’ original file for figure 4

Authors’ original file for figure 5

Authors’ original file for figure 6

Authors’ original file for figure 7

Authors’ original file for figure 8

Authors’ original file for figure 9

Authors’ original file for figure 10

Authors’ original file for figure 11

## Abbreviations

BCAR3: Breast cancer anti-estrogen resistance 3
DAPI: 4′,6-diamidino-2-phenylindole
DFS: Disease-free survival
DMEM: Dulbecco’s modified Eagle’s medium
DMFS: Distant metastasis-free survival
DMSO: Dimethyl sulfoxide
FBS: Fetal bovine serum
GEO: Gene Expression Omnibus
ER: Estrogen receptor
FBS: Fetal bovine serum
GOBO: Gene Expression-Based Outcome for Breast Cancer Online
MTT: 3-(4,5-dimethylthiazol-2-yl)-2,5-diphenyltetrazolium bromide (thiazolyl blue tetrazolium bromide)
NCBI: National Center for Biotechnology Information
OS: Overall survival
PBS: Phosphate-buffered saline
PR: Progesterone receptor
RFS: Relapse-free survival
RIPA: Radioimmunoprecipitation assay
siRNA: Small interfering RNA
TGFβ: Transforming growth factor β

## References

[1] Riggs BL, Hartmann LC (2003). Selective estrogen-receptor modulators—mechanisms of action and application to clinical practice. N Engl J Med.

[2] Shak S (1999). Overview of the trastuzumab (Herceptin) anti-HER2 monoclonal antibody clinical program in HER2-overexpressing metastatic breast cancer. Herceptin Multinational Investigator Study Group. Semin Oncol.

[3] Cornez N, Piccart MJ (2000). [Breast cancer and Herceptin] [Article in French]. Bull Cancer.

[4] Jordan VC (2000). How is tamoxifen’s action subverted?. J Natl Cancer Inst.

[5] van Agthoven T, van Agthoven TL, Dekker A, van der Spek PJ, Vreede L, Dorssers LC (1998). Identification of *BCAR*3 by a random search for genes involved in antiestrogen resistance of human breast cancer cells. EMBO J.

[6] Gotoh T, Cai D, Tian X, Feig LA, Lerner A (2000). p130^Cas^regulates the activity of AND-34, a novel Ral, Rap1, and R-Ras guanine nucleotide exchange factor. J Biol Chem.

[7] Schuh NR, Guerrero MS, Schrecengost RS, Bouton AH (2010). BCAR3 regulates Src/p130^Cas^association, Src kinase activity, and breast cancer adhesion signaling. J Biol Chem.

[8] Dorssers LCJ, van Agthoven T, Brinkman A, Veldscholte J, Smid M, Dechering KJ (2005). Breast cancer oestrogen independence mediated by *BCAR1* or *BCAR3*genes is transmitted through mechanisms distinct from the oestrogen receptor signalling pathway or the epidermal growth factor receptor signalling pathway. Breast Cancer Res.

[9] Riggins RB, Quilliam LA, Bouton AH (2003). Synergistic promotion of c-Src activation and cell migration by Cas and AND-34/BCAR3. J Biol Chem.

[10] van Agthoven T, Sieuwerts AM, Meijer-van Gelder ME, Look MP, Smid M, Veldscholte J, Sleijfer S, Foekens JA, Dorssers LC (2009). Relevance of breast cancer antiestrogen resistance genes in human breast cancer progression and tamoxifen resistance. J Clin Oncol.

[11] Massagué J (2008). TGFβ in cancer. Cell.

[12] Cao Y, Liu X, Zhang W, Deng X, Zhang H, Liu Y, Chen L, Thompson EA, Townsend CM, Ko TC (2009). TGF-β repression of Id2 induces apoptosis in gut epithelial cells. Oncogene.

[13] Cipriano R, Kan CE, Graham J, Danielpour D, Stampfer M, Jackson MW (2011). TGF-β signaling engages an ATM-CHK2-p53-independent RAS-induced senescence and prevents malignant transformation in human mammary epithelial cells. Proc Natl Acad Sci U S A.

[14] Ewen ME, Sluss HK, Whitehouse LL, Livingston DM (1993). TGFβ inhibition of Cdk4 synthesis is linked to cell cycle arrest. Cell.

[15] Smeland EB, Blomhoff HK, Holte H, Ruud E, Beiske K, Funderud S, Godal T, Ohlsson R (1987). Transforming growth factor type β (TGFβ) inhibits G_1_to S transition, but not activation of human B lymphocytes. Exp Cell Res.

[16] Kang Y, He W, Tulley S, Gupta GP, Serganova I, Chen CR, Manova-Todorova K, Blasberg R, Gerald WL, Massagué J (2005). Breast cancer bone metastasis mediated by the Smad tumor suppressor pathway. Proc Natl Acad Sci U S A.

[17] Muraoka RS, Dumont N, Ritter CA, Dugger TC, Brantley DM, Chen J, Easterly E, Roebuck LR, Ryan S, Gotwals PJ, Koteliansky V, Arteaga CL (2002). Blockade of TGF-β inhibits mammary tumor cell viability, migration, and metastases. J Clin Invest.

[18] Humbert L, Neel JC, Lebrun JJ (2010). Targeting TGFβ signaling in human cancer therapy. Trends Cell Mol Biol.

[19] Lebrun JJ (2012). The dual role of TGF in human cancer: from tumor suppression to cancer metastasis. ISRN Mol Biol.

[20] Xu J, Lamouille S, Derynck R (2009). TGF-β-induced epithelial to mesenchymal transition. Cell Res.

[21] Wilson EB, El-Jawhari JJ, Neilson AL, Hall GD, Melcher AA, Meade JL, Cook GP (2011). Human tumour immune evasion via TGF-β blocks NK cell activation but not survival allowing therapeutic restoration of anti-tumour activity. PLoS One.

[22] Flavell RA, Sanjabi S, Wrzesinski SH, Licona-Limón P (2010). The polarization of immune cells in the tumour environment by TGFβ. Nat Rev Immunol.

[23] Massagué J, Gomis RR (2006). The logic of TGFβ signaling. FEBS Lett.

[24] Shi Y, Massagué J (2003). Mechanisms of TGF-β signaling from cell membrane to the nucleus. Cell.

[25] Padua D, Massagué J (2009). Roles of TGFβ in metastasis. Cell Res.

[26] Dai M, Al-Odaini AA, Arakelian A, Rabbani SA, Ali S, Lebrun JJ (2012). A novel function for p21Cip1 and acetyltransferase p/CAF as critical transcriptional regulators of TGFβ-mediated breast cancer cell migration and invasion. Breast Cancer Res.

[27] Neve RM, Chin K, Fridlyand J, Yeh J, Baehner FL, Fevr T, Clark L, Bayani N, Coppe JP, Tong F, Speed T, Spellman PT, DeVries S, Lapuk A, Wang NJ, Kuo WL, Stilwell JL, Pinkel D, Albertson DG, Waldman FM, McCormick F, Dickson RB, Johnson MD, Lippman M, Ethier S, Gazdar A, Gray JW (2006). A collection of breast cancer cell lines for the study of functionally distinct cancer subtypes. Cancer Cell.

[28] Fils-Aimé N, Dai M, Guo J, El-Mousawi M, Kahramangil B, Neel JC, Lebrun JJ (2013). MicroRNA-584 and the protein phosphatase and actin regulator 1 (PHACTR1), a new signaling route through which transforming growth factor-β mediates the migration and actin dynamics of breast cancer cells. J Biol Chem.

[29] Suzuki K, Bose P, Leong-Quong RY, Fujita DJ, Riabowol K (2010). REAP: a two minute cell fractionation method. BMC Res Notes.

[30] Makkinje A, Near RI, Infusini G, Vanden Borre P, Bloom A, Cai D, Costello CE, Lerner A (2009). AND-34/BCAR3 regulates adhesion-dependent p130Cas serine phosphorylation and breast cancer cell growth pattern. Cell Signal.

[31] Near RI, Zhang Y, Makkinje A, Vanden Borre P, Lerner A (2007). AND-34/BCAR3 differs from other NSP homologs in induction of anti-estrogen resistance, cyclin D1 promoter activation and altered breast cancer cell morphology. J Cell Physiol.

[32] Ringnér M, Fredlund E, Häkkinen J, Borg Å, Staaf J (2011). GOBO: gene expression-based outcome for breast cancer online. PLoS One.

[33] Györffy B, Lanczky A, Eklund AC, Denkert C, Budczies J, Li Q, Szallasi Z (2010). An online survival analysis tool to rapidly assess the effect of 22,277 genes on breast cancer prognosis using microarray data of 1,809 patients. Breast Cancer Res Treat.

[34] Ma XJ, Wang Z, Ryan PD, Isakoff SJ, Barmettler A, Fuller A, Muir B, Mohapatra G, Salunga R, Tuggle JT, Tran Y, Tran D, Tassin A, Amon P, Wang W, Enright E, Stecker K, Estepa-Sabal E, Smith B, Younger J, Balis U, Michaelson J, Bhan A, Habin K, Baer TM, Brugge J, Haber DA, Erlander MG, Sgroi DC (2004). A two-gene expression ratio predicts clinical outcome in breast cancer patients treated with tamoxifen. Cancer Cell.

[35] Sims D, Bursteinas B, Gao Q, Jain E, MacKay A, Mitsopoulos C, Zvelebil M (2010). ROCK: a breast cancer functional genomics resource. Breast Cancer Res Treat.

[36] Chin K, DeVries S, Fridlyand J, Spellman PT, Roydasgupta R, Kuo WL, Lapuk A, Neve RM, Qian Z, Ryder T, Chen F, Feiler H, Tokuyasu T, Kingsley C, Dairkee S, Meng Z, Chew K, Pinkel D, Jain A, Ljung BM, Esserman L, Albertson DG, Waldman FM, Gray JW (2006). Genomic and transcriptional aberrations linked to breast cancer pathophysiologies. Cancer Cell.

[37] Wendt MK, Smith JA, Schiemann WP (2009). p130Cas is required for mammary tumor growth and transforming growth factor-β-mediated metastasis through regulation of Smad2/3 activity. J Biol Chem.

[38] Kim W, Kang YS, Soo Kim J, Shin NY, Hanks SK, Song WK (2008). The integrin-coupled signaling adaptor p130Cas suppresses Smad3 function in transforming growth factor-β signaling. Mol Biol Cell.

[39] Cai D, Iyer A, Felekkis KN, Near RI, Luo Z, Chernoff J, Albanese C, Pestell RG, Lerner A (2003). AND-34/BCAR3, a GDP exchange factor whose overexpression confers antiestrogen resistance, activates Rac, PAK1, and the cyclin D1 promoter. Cancer Res.

[40] Flanagan L, Van Weelden K, Ammerman C, Ethier SP, Welsh J (1999). SUM-159PT cells: a novel estrogen independent human breast cancer model system. Breast Cancer Res Treat.

[41] Minn AJ, Kang Y, Serganova I, Gupta GP, Giri DD, Doubrovin M, Ponomarev V, Gerald WL, Blasberg R, Massagué J (2005). Distinct organ-specific metastatic potential of individual breast cancer cells and primary tumors. J Clin Invest.

[42] Kucich U, Rosenbloom JC, Herrick DJ, Abrams WR, Hamilton AD, Sebti SM, Rosenbloom J (2001). Signaling events required for transforming growth factor-β stimulation of connective tissue growth factor expression by cultured human lung fibroblasts. Arch Biochem Biophys.

[43] Brunschwig EB, Wilson K, Mack D, Dawson D, Lawrence E, Willson JK, Lu S, Nosrati A, Rerko RM, Swinler S, Beard L, Lutterbaugh JD, Willis J, Platzer P, Markowitz S (2003). *PMEPA1*, a transforming growth factor-β-induced marker of terminal colonocyte differentiation whose expression is maintained in primary and metastatic colon cancer. Cancer Res.

[44] Stopa M, Anhuf D, Terstegen L, Gatsios P, Gressner AM, Dooley S (2000). Participation of Smad2, Smad3, and Smad4 in transforming growth factor β (TGF-β)-induced activation of Smad7: the TGF-β response element of the promoter requires functional Smad binding element and E-box sequences for transcriptional regulation. J Biol Chem.

[45] Mazars P, Barboule N, Baldin V, Vidal S, Ducommun B, Valette A (1995). Effects of TGF-β1 (transforming growth factor-β1) on the cell cycle regulation of human breast adenocarcinoma (MCF-7) cells. FEBS Lett.

[46] Trinh BQ, Barengo N, Naora H (2011). Homeodomain protein DLX4 counteracts key transcriptional control mechanisms of the TGF-β cytostatic program and blocks the antiproliferative effect of TGF-β. Oncogene.

[47] Lamouille S, Connolly E, Smyth JW, Akhurst RJ, Derynck R (2012). TGF-β-induced activation of mTOR complex 2 drives epithelial-mesenchymal transition and cell invasion. J Cell Sci.

[48] Giampieri S, Manning C, Hooper S, Jones L, Hill CS, Sahai E (2009). Localized and reversible TGFβ signalling switches breast cancer cells from cohesive to single cell motility. Nat Cell Biol.

[49] Lamouille S, Derynck R (2007). Cell size and invasion in TGF-β-induced epithelial to mesenchymal transition is regulated by activation of the mTOR pathway. J Cell Biol.

[50] Chhabra ES, Higgs HN (2007). The many faces of actin: matching assembly factors with cellular structures. Nat Cell Biol.

[51] Safina A, Ren MQ, Vandette E, Bakin AV (2008). TAK1 is required for TGF-β1-mediated regulation of matrix metalloproteinase-9 and metastasis. Oncogene.

[52] Meulmeester E, Ten Dijke P (2011). The dynamic roles of TGF-β in cancer. J Pathol.

[53] Schrecengost RS, Riggins RB, Thomas KS, Guerrero MS, Bouton AH (2007). Breast cancer antiestrogen resistance-3 expression regulates breast cancer cell migration through promotion of p130^Cas^membrane localization and membrane ruffling. Cancer Res.

[54] Ho J, Cocolakis E, Dumas VM, Posner BI, Laporte SA, Lebrun JJ (2005). The G protein-coupled receptor kinase-2 is a TGFβ-inducible antagonist of TGFβ signal transduction. EMBO J.

[55] Ho J, Chen H, Lebrun JJ (2007). Novel dominant negative Smad antagonists to TGFβ signaling. Cell Signal.

