# Supplementary figures and images for: Breast cancer anti-estrogen resistance 3 inhibits transforming growth factor β/Smad signaling and associates with favorable breast cancer disease outcomes
Source: Breast Cancer Res. 2014 Dec 13;16:476. doi: 10.1186/s13058-014-0476-9 (PMC4311507; doi:10.1186/s13058-014-0476-9)

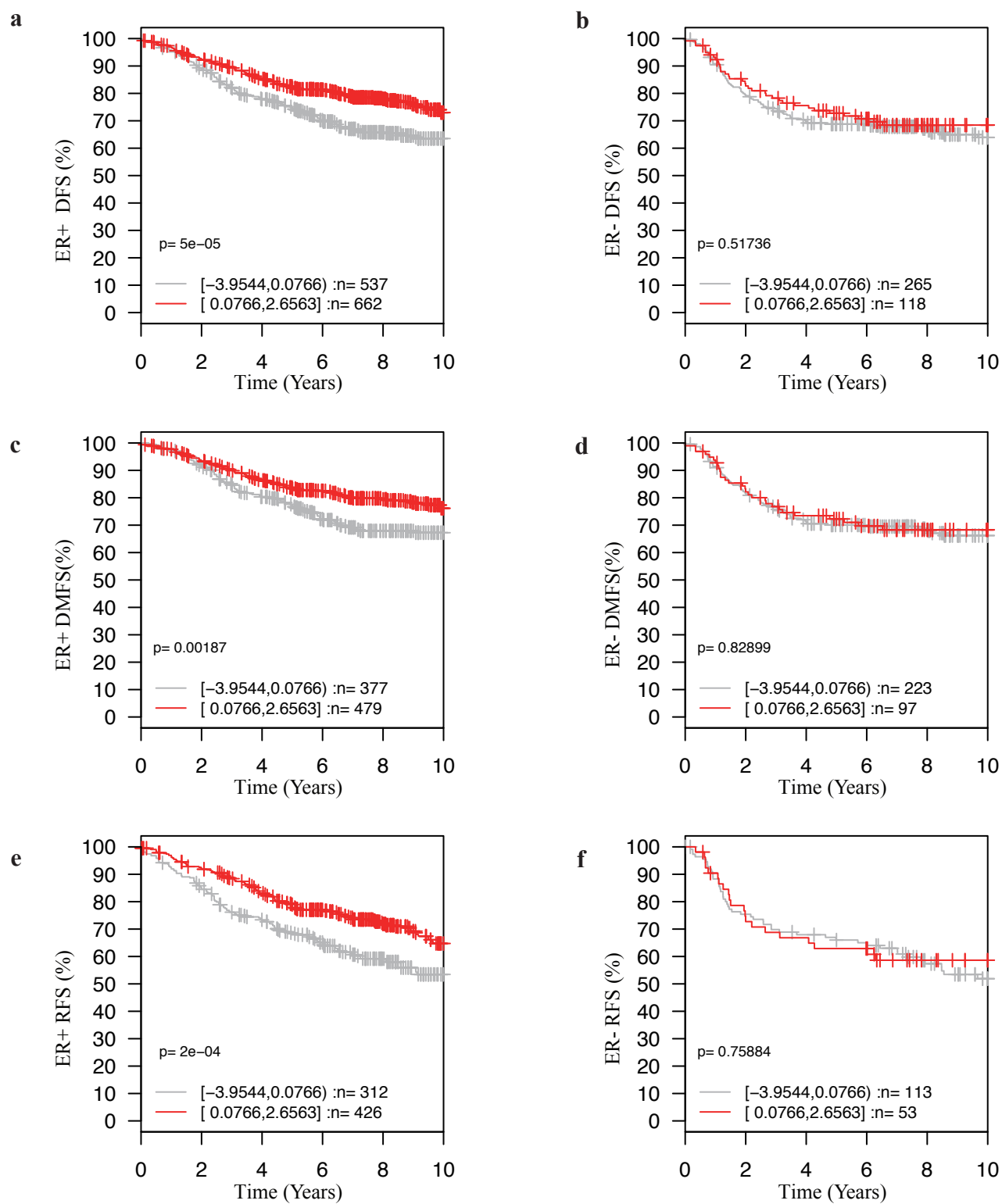

Supplement: Supplementary file 1 — Additional file 1: Figure S1.: Low BCAR3 expression predicts poor prognosis in ER+ breast cancer. (a) and (b) Kaplan-Meier survival curved generated by GOBO gene expression-based outcome tool, showing status of disease-free survival (DFS) of ER+ breast cancer patients (a) and ER− patients (b). Patients were separated by median of signal intensity from an Affymetrix probe targeting BCAR3 (204032_at) in microarray analysis. Survival data of the high-expression group are shown by the red curve, and those of the low-expression group are shown by the gray curve. (c) and (d) Kaplan-Meier survival curves showing status of distant metastasis-free survival (DMFS) of ER+ patients (c) and ER− patients (d). Patients were separated by median of signal intensity from 204032_at. (e) and (f) Kaplan-Meier survival curves showing status of relapse-free survival (RFS) of ER+ patients (e) and ER− patients (f). (PDF 458 KB) [file 13058_2014_476_MOESM1_ESM.pdf]

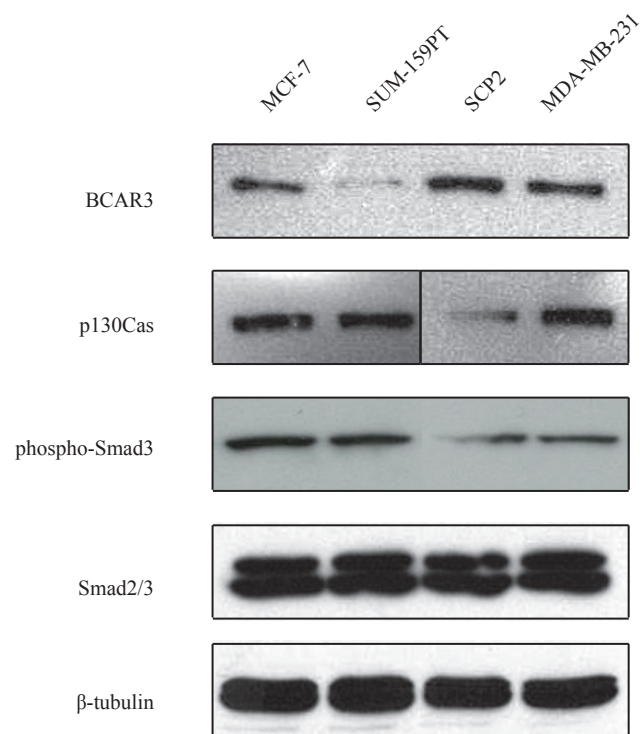

Supplement: Supplementary file 2 — Additional file 2: Figure S2.: Protein levels of phospho-Smad3 and Smad2/3 in selected breast cancer cells. Levels of phospho-Smad3, Smad2/3, BCAR3 and p130Cas in total cell lysates of MCF-7, SUM-159PT, SCP2 and MDA-MB-231 cells were determined by Western blot analysis. (PDF 469 KB) [file 13058_2014_476_MOESM2_ESM.pdf]

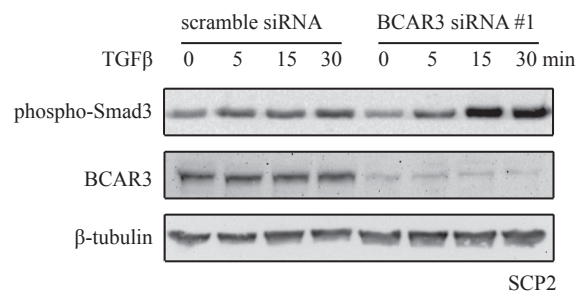

Supplement: Supplementary file 3 — Additional file 3: Figure S3.: Knocking down BCAR3 enhances TGFβ-induced Smad3 phosphorylation in SCP2 cells. SCP2 cells were transfected with BCAR3 siRNA ((#1: SASI_Hs01_00236261), starved overnight and stimulated with 200 pM TGFβ 48 hours poststarvation for the indicated time periods. Levels of phospho-Smad3 and BCAR3 were examined by Western blot analysis. (PDF 446 KB) [file 13058_2014_476_MOESM3_ESM.pdf]

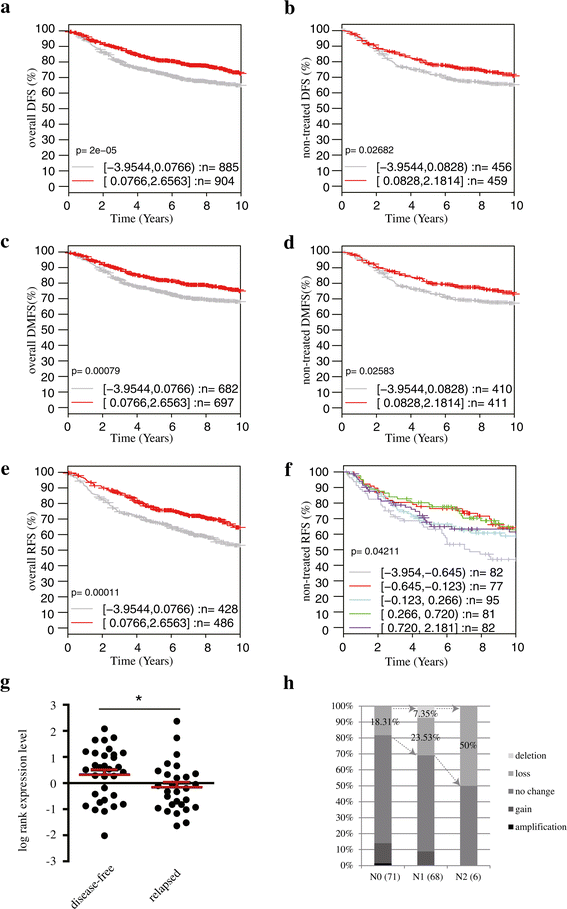

Supplement: Supplementary file 4 — Authors’ original file for figure 1 [file 13058_2014_476_MOESM4_ESM.gif]

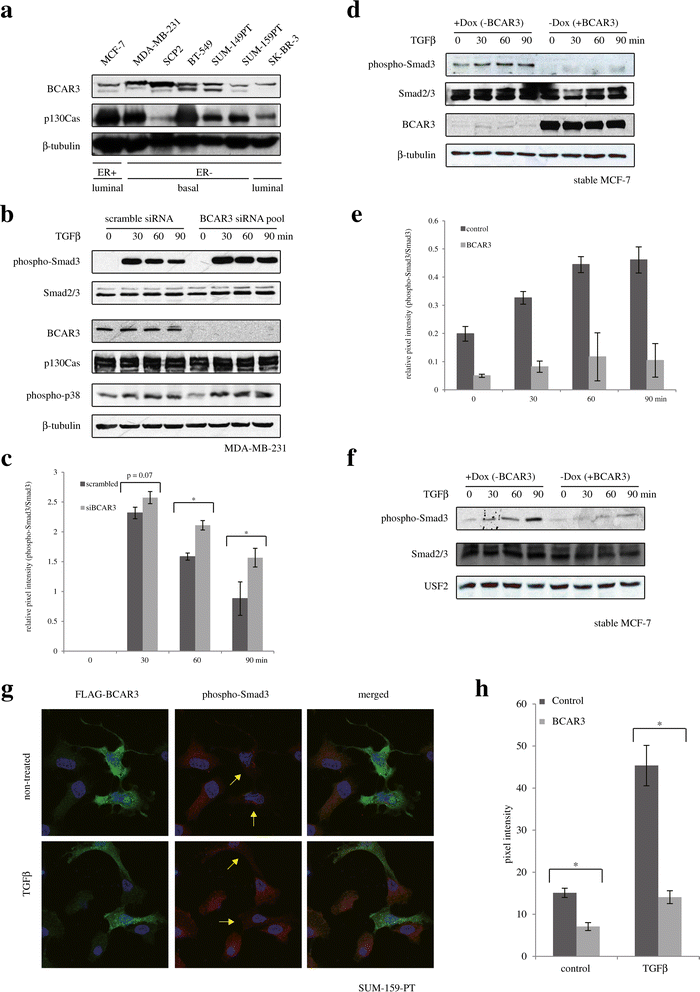

Supplement: Supplementary file 5 — Authors’ original file for figure 2 [file 13058_2014_476_MOESM5_ESM.gif]

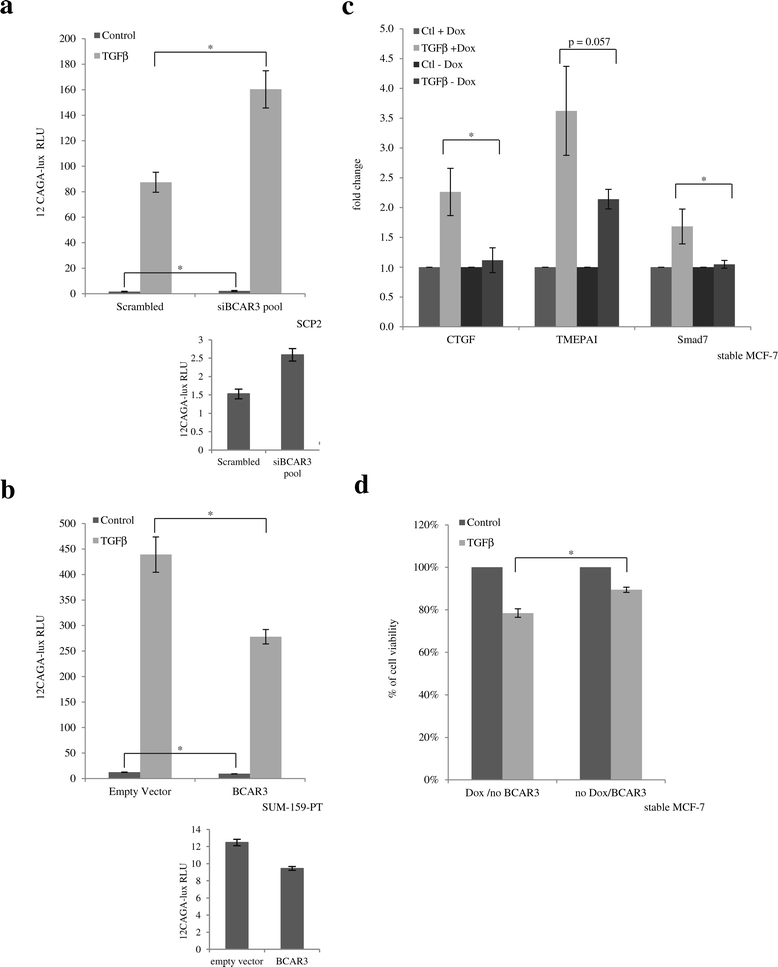

Supplement: Supplementary file 6 — Authors’ original file for figure 3 [file 13058_2014_476_MOESM6_ESM.gif]

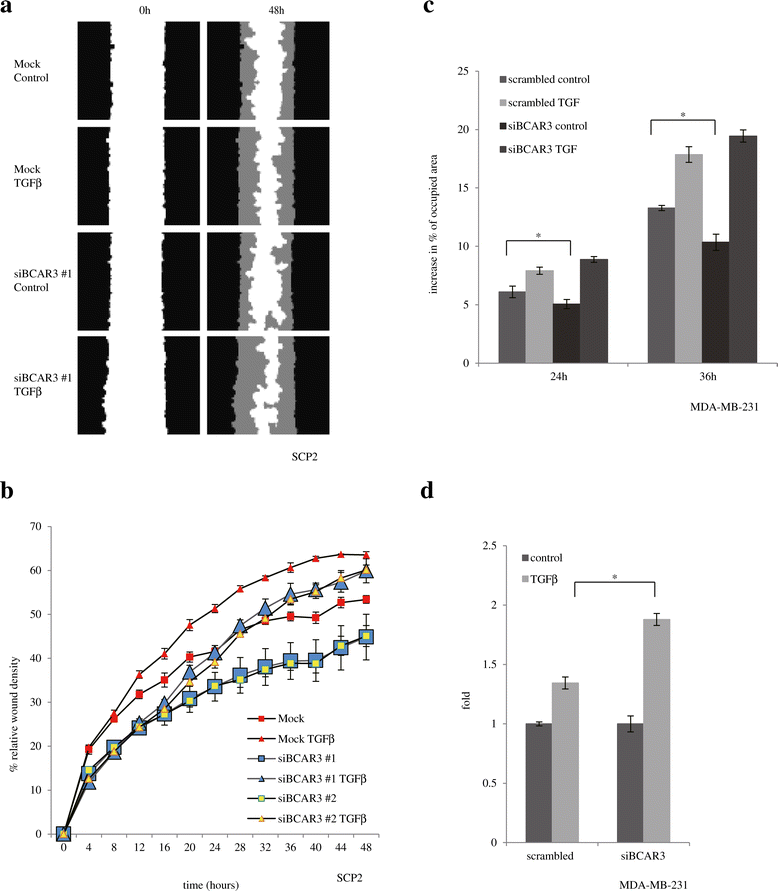

Supplement: Supplementary file 7 — Authors’ original file for figure 4 [file 13058_2014_476_MOESM7_ESM.gif]

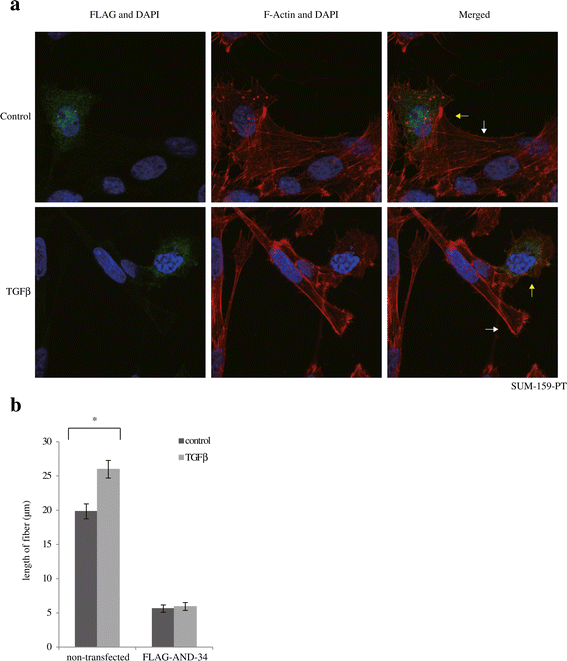

Supplement: Supplementary file 8 — Authors’ original file for figure 5 [file 13058_2014_476_MOESM8_ESM.gif]

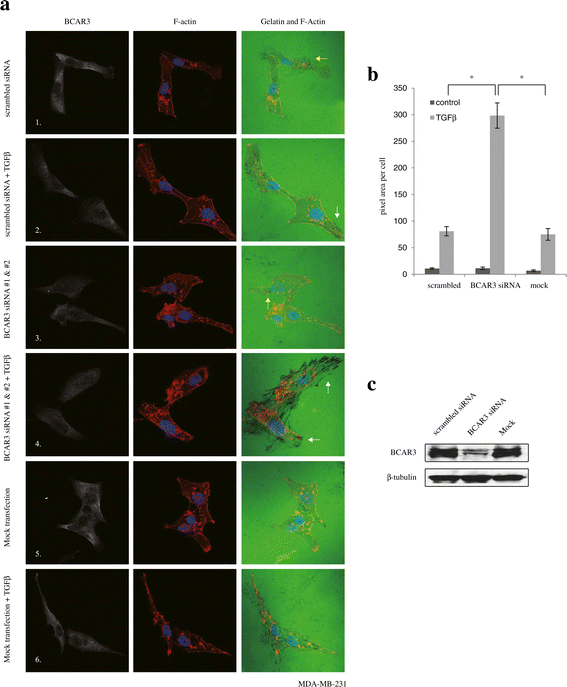

Supplement: Supplementary file 9 — Authors’ original file for figure 6 [file 13058_2014_476_MOESM9_ESM.gif]

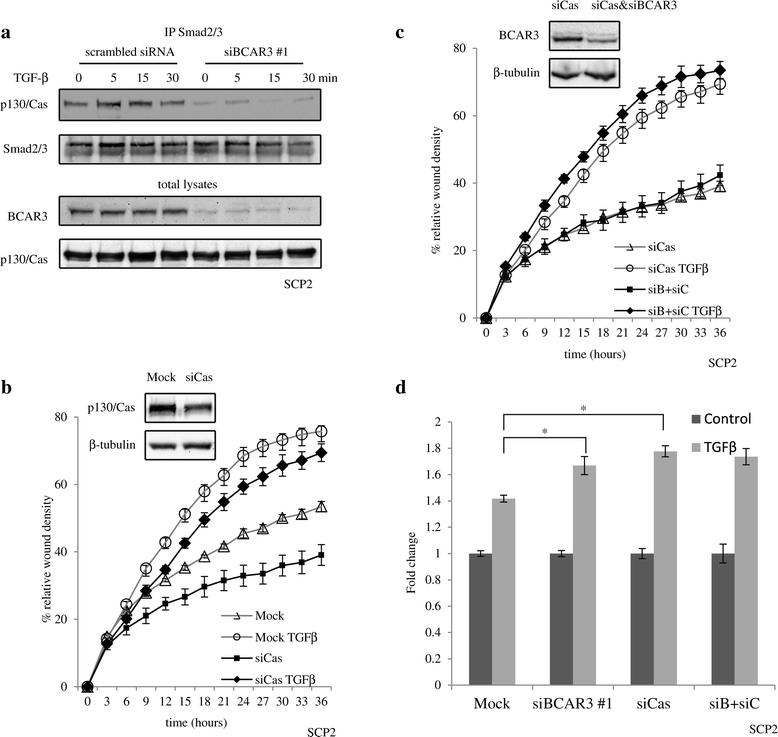

Supplement: Supplementary file 10 — Authors’ original file for figure 7 [file 13058_2014_476_MOESM10_ESM.gif]

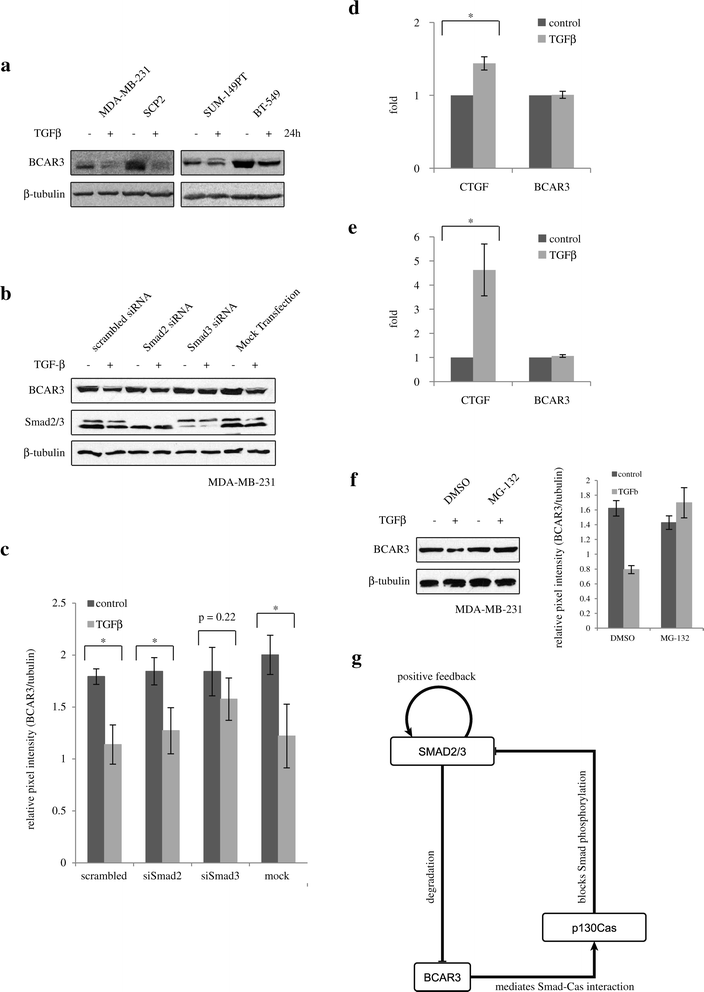

Supplement: Supplementary file 11 — Authors’ original file for figure 8 [file 13058_2014_476_MOESM11_ESM.gif]
